# Echinocandins Localized to the Target-Harboring Cell Surface Are Not Degraded but Those Entering the Vacuole Are

**DOI:** 10.1021/acschembio.2c00060

**Published:** 2022-04-11

**Authors:** Qais Z. Jaber, Dana Logviniuk, Adi Yona, Micha Fridman

**Affiliations:** School of Chemistry, Raymond & Beverly Sackler Faculty of Exact Sciences, Tel Aviv University, Tel Aviv 6997801, Israel

## Abstract

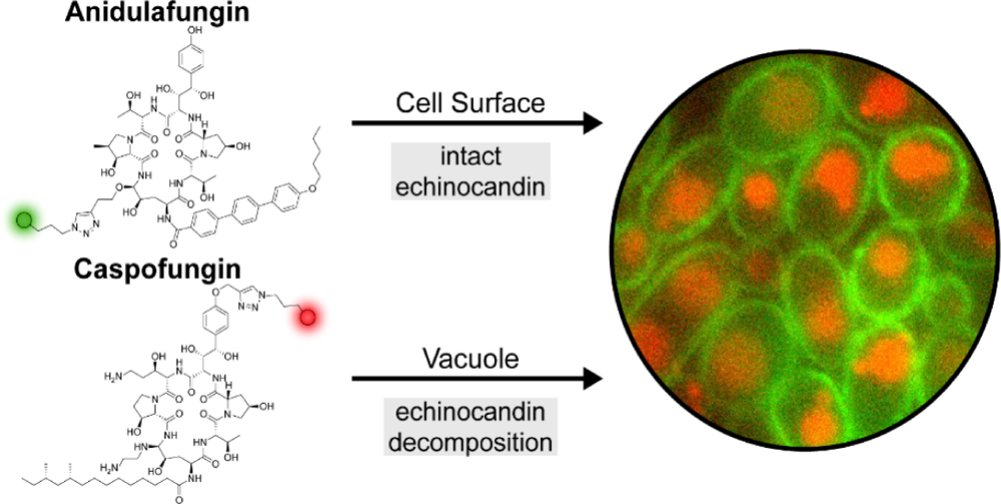

Echinocandin antifungal drugs have a broad spectrum of activities and excellent safety profiles. These agents noncompetitively inhibit the formation of the major polysaccharide component of the fungal cell wall, a reaction catalyzed by the membrane-bound β-glucan synthase (GS) protein complex. We have developed fluorescent probes of three echinocandin drugs: caspofungin (CSF), anidulafungin (ANF), and rezafungin (RZF). Fluorescent echinocandins had the same spectrum of activities as the parent echinocandins, supporting the fact that conjugation of the dye did not alter their mode of action. Of the three echinocandins, ANF has the most potent in vitro activity. Investigation of the subcellular distribution of the fluorescent echinocandins in live *Candida* yeast cells revealed that despite their high structural similarity, each of the drug probes had a unique subcellular distribution pattern. Fluorescent CSF, which is the least potent of the three echinocandins, accumulated in *Candida* vacuoles; fluorescent ANF localized in the extracellular environment and on the yeast cell surface where the target GS resides; and fluorescent RZF was partitioned between the surface and the vacuole over time. Recovery of fluorescent CSF from *Candida* cells revealed substantial degradation over time; functional vacuoles were necessary for this degradation. Under the same conditions, fluorescent ANF was not degraded. This study supports the “target-oriented drug subcellular localization” principle. In the case of echinocandins, localization to the cell surface can contribute to improved potency and accumulation in vacuoles induces degradation leading to drug deactivation.

## Introduction

The incidence of fungal infections has risen sharply during the last few decades due to rising numbers of immunosuppressed patients and the increasing prevalence of intrinsically drug-resistant and drug-tolerant fungal pathogens.^1−3^ There is a limited number of clinically approved antifungal drugs, and the most commonly used belong to just four classes: polyenes, azoles, allylamines, and echinocandins.^4^

**Figure 1:**
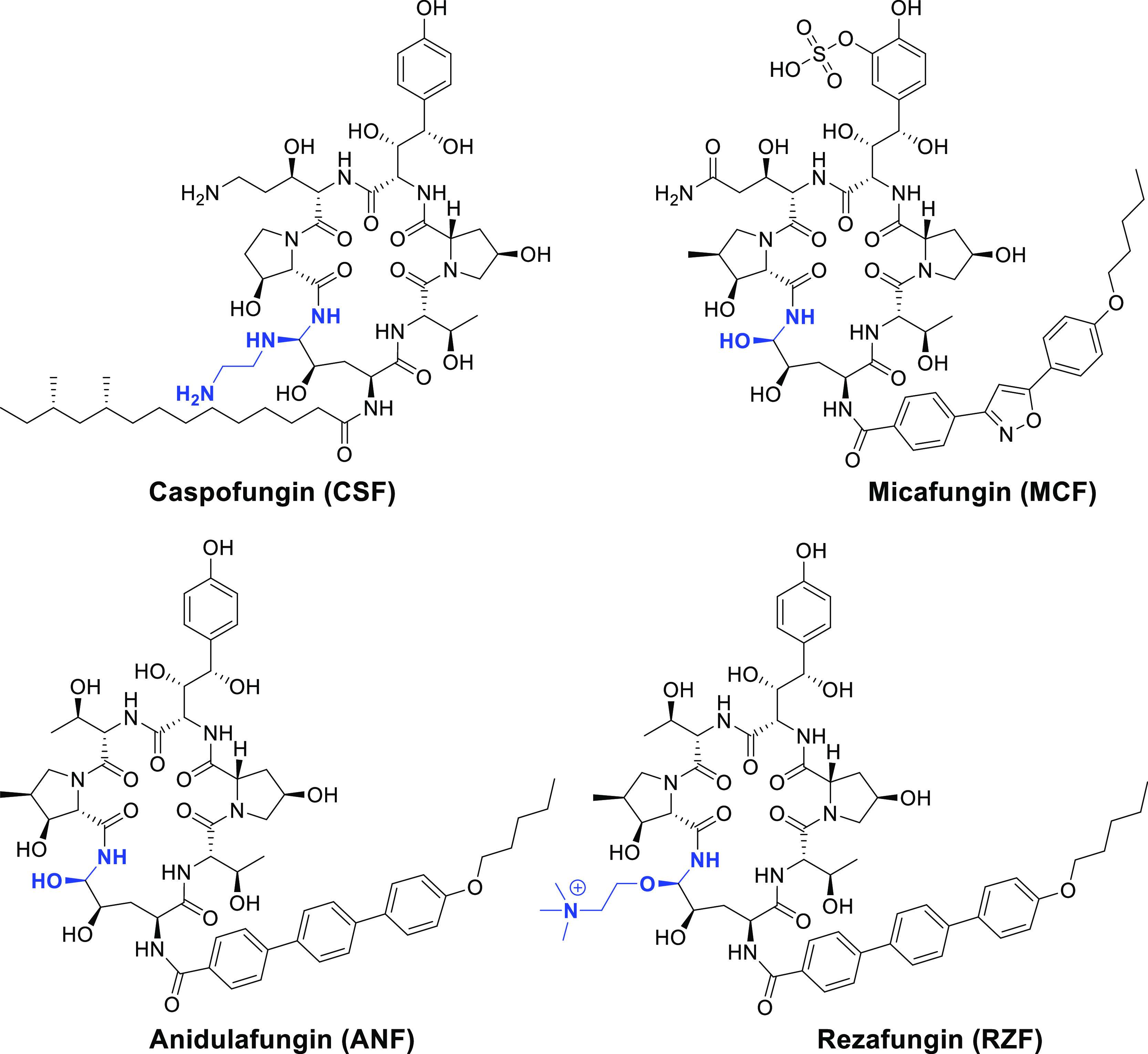
Structures of echinocandin drugs in clinical use (CSF, MCF, and ANF) and in clinical development (RZF). Aminal, hemiaminal, and choline hemiaminal ether functionalities are colored in blue.

Echinocandins are the most recently developed class of antifungals.^4,5^ In January 2001, caspofungin (CSF), developed by Merck & Co., was the first echinocandin to receive the Food and Drug Administrations’ approval (Figure 1). Micafungin (MCF), developed by Astellas Pharma Inc., was approved in 2005 and anidulafungin (ANF), developed by Pfizer, in 2006.^6−8^ Echinocandins have rapidly become drugs of choice for first-line treatment of candidemia and invasive candidiasis, severe systemic infections caused by pathogenic yeast of the genus *Candida*, the most prevalent fungal pathogen in humans.^9,10^

Echinocandins have limited chemical stability and solubility and are administered by a daily infusion.^11−13^ The peptide ring of CSF consists of an aminal functional group, and those of MCF and ANF contain a hemiaminal (Figure 1). Both aminal and hemiaminal functionalities are susceptible to hydrolysis in aqueous solutions, and the stabilities of the three echinocandins in clinical use are pH-dependent. At physiological pH, chemical degradation of echinocandins is initiated with a ring-opening event in which the aminal or hemiaminal linkage is hydrolyzed, resulting in the formation of a linear peptide with a terminal amide and a terminal aldehyde.^14−17^

Intending to develop echinocandins with improved chemical stability under physiological conditions, Cidara Therapeutics developed rezafungin (RZF), a novel ANF derivative with distinctive pharmacokinetic and pharmacodynamic profiles, which entered phase III trials in October 2018 (Figure 1).^18−21^ RZF is generated from ANF via a single-step conversion of the hemiaminal of ANF to a choline-based hemiaminal ether that is more stable than the hemiaminal in ANF.^22^ Instead of the daily intravenous administration regime used for the other three echinocandins, RZF can be administered intravenously once a week.^23^ The significantly prolonged efficacy is due to enhanced stability: Following incubation in human plasma for 44 h at 37 °C, the percent of intact RZF was 91%, whereas that of ANF was 7%.^18^

Echinocandins interfere with the biosynthesis of β-glucan, an essential fungal cell wall polysaccharide, by non-competitively inhibiting the activity of β-glucan synthase (GS).^24,25^ GS is a membrane-bound protein complex that catalyzes the formation of a polysaccharide composed of β-(1 → 3)-linked glucose units.^26^ The GS catalytic subunit is the Fks, an integral membrane glycosyl transferase.^27,28^ Echinocandins are thought to bind to the extracellular domain of Fks, and mutations in its extracellular domains, so-called “hotspot” regions, are associated with resistance to these drugs.^24,29^ The GS complex has been validated as the target of echinocandins by both biochemical and genetic approaches; however, the subcellular distributions of echinocandins, their mechanisms of entry into fungal cells, and the relationship between their uptake and antifungal activity are not fully understood.^30,31^

**Scheme 1:**
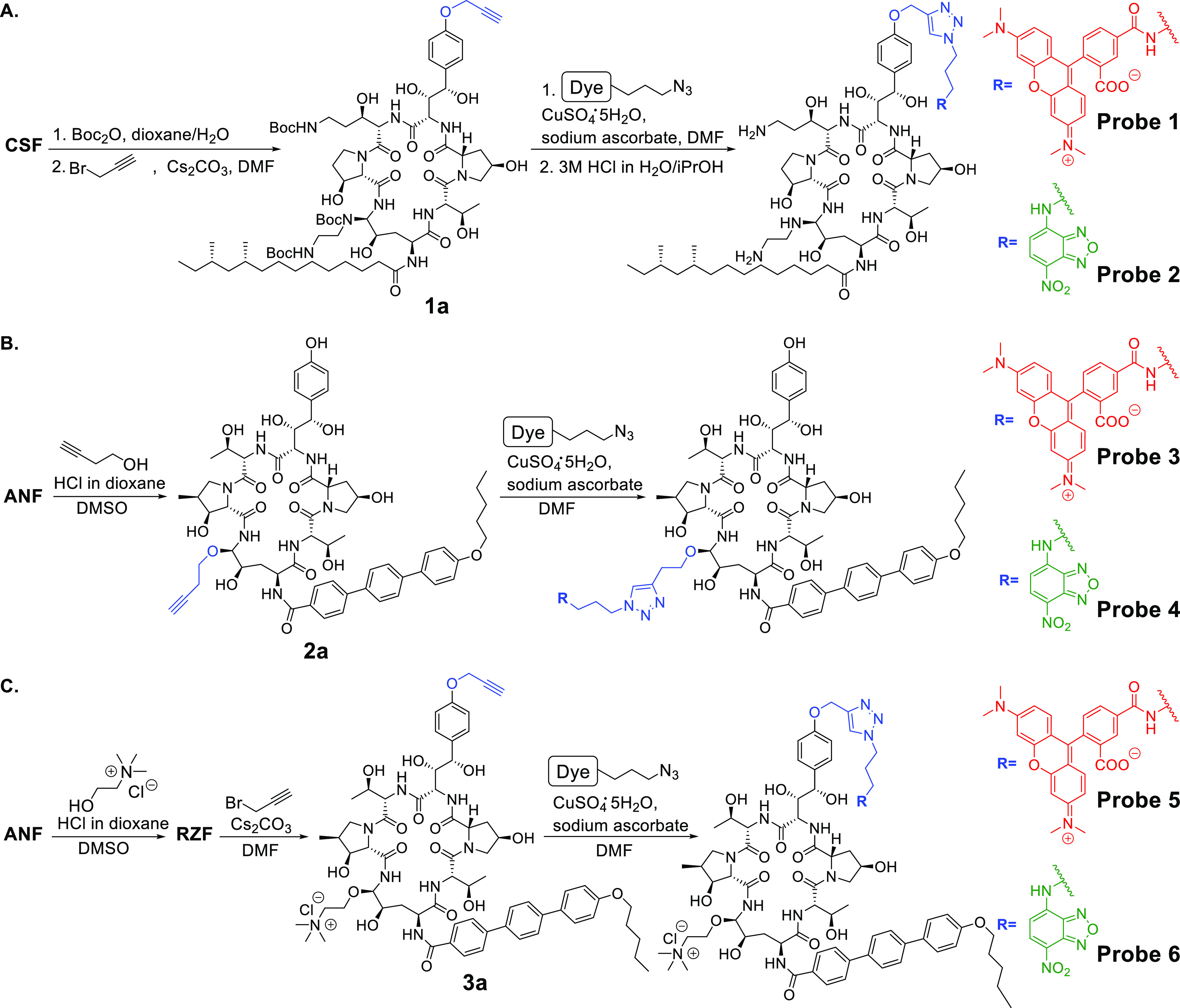
Synthesis and Structures of Fluorescent CSF Probes (**1** and **2**), Fluorescent ANF Probes (**3** and **4**), and Fluorescent RZF Probes (**5** and **6**). The echinocandin drug scaffold is colored black, linker units are colored blue, TMR dye is colored red, and NBD dye is colored green.

We recently synthesized a fluorescently labeled derivative of CSF (Probe **1**, Scheme 1) and discovered that it is readily taken into *Candida* cells where it accumulates in the vacuole via endocytosis shortly after introduction.^32^ This indicates that a significant percentage of this echinocandin does not accumulate at the cell surface, the location of its target, which may impair its efficacy.^32^ On average, the level of uptake of the fluorescently labeled CSF was significantly higher in echinocandin-resistant *Candida* strains than in echinocandin-susceptible strains.^32^

Comparison of minimal inhibitory concentration (MIC) values of CSF to those of ANF indicates that the latter has more potent antifungal activity in vitro.^33,34^ The abilities of the three clinically used echinocandins to inhibit the catalytic activity of the GS complex were previously determined using fractions containing partially purified GS, which were obtained through product entrapment from a collection of resistant and susceptible strains.^33^ The half-maximal inhibitory concentration (IC_50_) values of CSF and ANF depend on the strain from which the GS is isolated. The measured IC_50_ values of CSF were similar to or up to sevenfold higher than those of ANF. These differences did not always correlate with the differences in MIC values, suggesting that additional parameters contribute to differences in antifungal activities of these drugs.

In search of design guidelines for the next generation of echinocandin drugs, we have evaluated how specific molecular characteristics of three members of the echinocandin class of antifungals affect their subcellular distribution patterns and antifungal efficacies. We have previously reported on the development of fluorescent probes for studying the effects of antifungal azoles and antifungal cationic amphiphiles against cells of pathogenic fungi.^35−40^ In this study, we applied a similar strategy and designed and synthesized fluorescent probes of CSF, ANF, and RZF and studied their time-dependent subcellular distribution and stability in live yeast cells of the genus *Candida*.

## Results and Discussion

### Design and Synthesis of Fluorescent Echinocandin Probes

To facilitate tracking of the echinocandins in live yeast cells, each drug was conjugated to two different fluorescent dyes that differ significantly in their molecular weight and formula: tetramethylrhodamine (TMR absorption 555 nm, emission 585 nm; red in Scheme 1) and nitrobenzoxadiazole (NBD, absorption 470 nm, emission 540 nm; green in Scheme 1). We reasoned that since NBD is considerably smaller than TMR, comparison of the subcellular distributions of echinocandins that differ solely by the conjugated fluorescent dye would reveal whether the dye affects the subcellular distribution and antifungal activity of the drug.

CSF probes were prepared by selective conjugation of the propargyl group to the phenol of the 3*S*,4*S*-dihydroxy-l-homotyrosine amino acid of CSF to afford the alkyne-functionalized CSF derivative **1a**, which was then coupled to the azide-functionalized fluorescent dye (TMR or NBD) using a click reaction to form fluorescent CSF probes **1** and **2** (Scheme 1A) as we previously reported.^32^ For the preparation of fluorescent ANF probes **3** and **4**, ANF was reacted with 3-butynyl alcohol under acidic conditions to generate the corresponding 3-butynyl-hemiaminal ether derivative **2a**, which was then coupled to the azide-functionalized fluorescent dye using a click reaction to form fluorescent probes **3** and **4** (Scheme 1B). RZF was prepared from ANF through etherification of the hemiaminal group with choline under acidic conditions according to a procedure developed by Cidara Therapeutics,^22^ followed by propargylation of its 3*S*,4*S*-dihydroxy-l-homotyrosine phenol to form the corresponding functionalized echinocandin **3a** (Scheme 1C). The two RZF fluorescent probes **5** and **6** were then generated using click reactions between azide-functionalized fluorescent dyes and the propargyl-functionalized RZF derivative (Scheme 1C).

### The TMR- and NBD-labeled Echinocandin Probes Have the Same Spectrum of Antifungal Activity as the Parent Drugs

To investigate the suitability of fluorescent probes **1**–**6** as indicators of the subcellular distribution of the parent echinocandin drugs, we first evaluated their antifungal activity against echinocandin-susceptible and echinocandin-resistant yeast strains. The antifungal activities of fluorescent echinocandin probes **1**–**6** were evaluated against a panel of 14 *Candida* strains and compared to those of the three echinocandin drugs from which they were derived.

**Figure 2:**
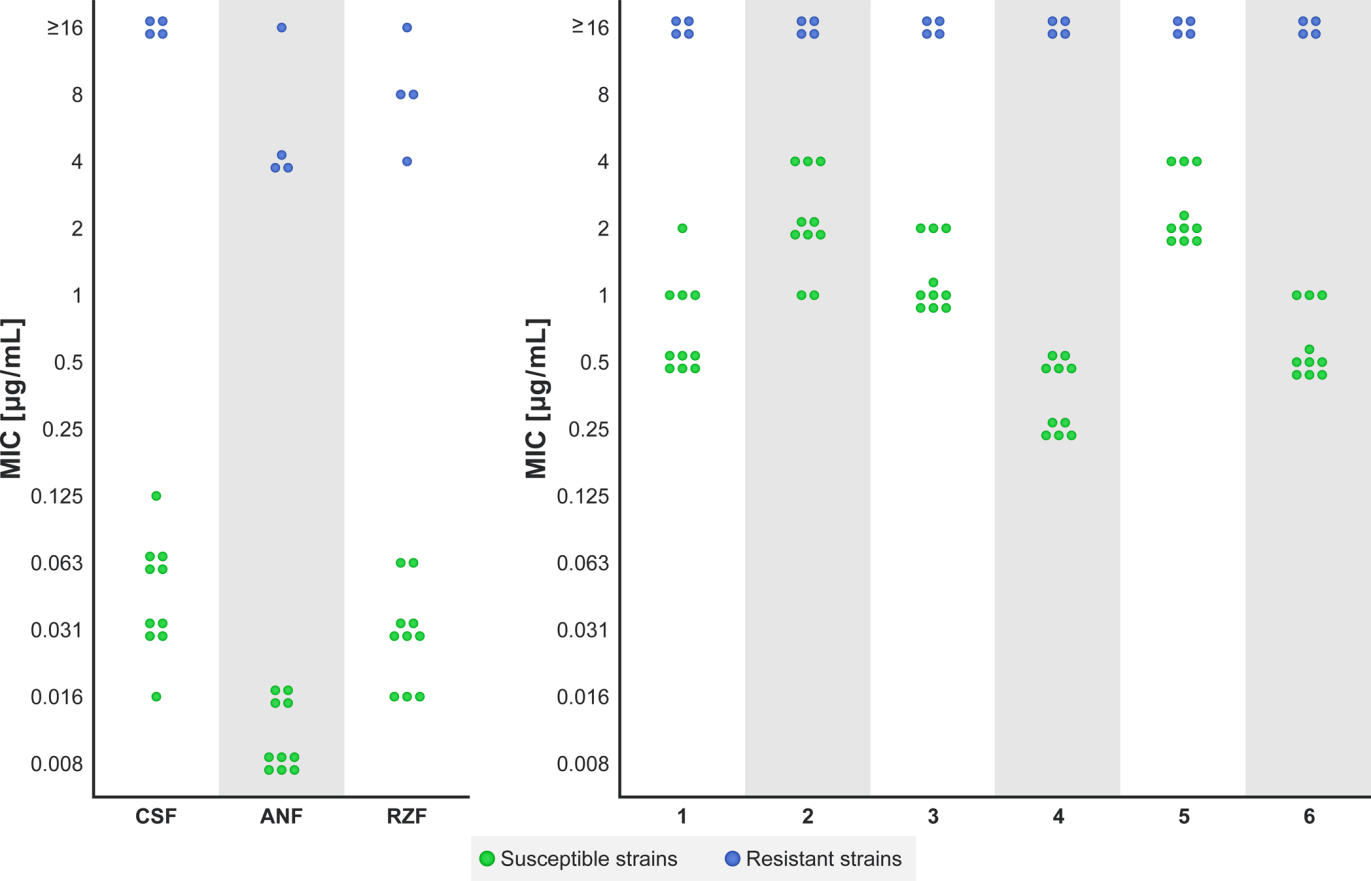
Antifungal activities of parent CSF, ANF, and RZF drugs (left panel) and fluorescent probes **1**–**6** (right panel). Echinocandin-susceptible strains are colored green and echinocandin-resistant strains are colored blue. MIC values were determined as the lowest concentration that caused ≥75% decrease in growth as measured at OD_600_. Results were reproduced in at least two independent experiments.

To confirm that, similar to the parent echinocandins, the fluorescent probes **1**–**6** target the Fks catalytic subunits of the GS complex, the panel in this study included *Candida albicans* and *Candida glabrata* strains composed of two subsets of echinocandin-susceptible strains and their corresponding isogenic echinocandin-resistant strains. The resistant strains have point mutations within or near the defined hotspots in the *FKS1* and/or *FKS2* genes of the GS complex that confer resistance to echinocandins (Table S1). The MIC values for all strains, determined using the broth double-dilution method, are summarized in Figure 2 and Table S2.

In general, labeling of ANF and RZF with NBD resulted in slightly superior antifungal activities relative to the corresponding TMR-labeled probes. In contrast, the antifungal activity of the TMR-labeled CSF probe was slightly superior to that of the corresponding NBD-labeled probe. For the conjugation of the fluorescent dye, CSF and RZF probes were functionalized at the same position, the 3*S*,4*S*-dihydroxy-l-homotyrosine phenol. The antifungal activity of the TMR-labeled CSF probe **1** was superior to that of the NBD-labeled CSF probe **2**, whereas the activity of the NBD-labeled RZF probe **6** was superior to that of the corresponding TMR-labeled RZF probe **5**. These results indicate that the optimal choice of fluorescent dye depends on the individual echinocandin and that the molecular weight of the fluorescent dye cannot serve as a general guideline for the design of fluorescent echinocandin probes with optimal antifungal activity.

Importantly, all of the fluorescent echinocandin probes had the same spectra of activity as their corresponding parent echinocandin drugs. The echinocandin-resistant strains, in which resistance is due to mutations in the *FKS1* and/or *FKS2* genes of the GS complex, were resistant to these fluorescent probes, and echinocandin-susceptible strains were susceptible. This supports the hypothesis that similar to the echinocandins from which they were derived, probes **1**–**6** inhibit the activity of the GS complex.

### Live-Cell Imaging Reveals Time-Dependent Differences in the Distribution of Probes Between the Surface and Vacuoles of Yeast Cells

**Figure 3:**
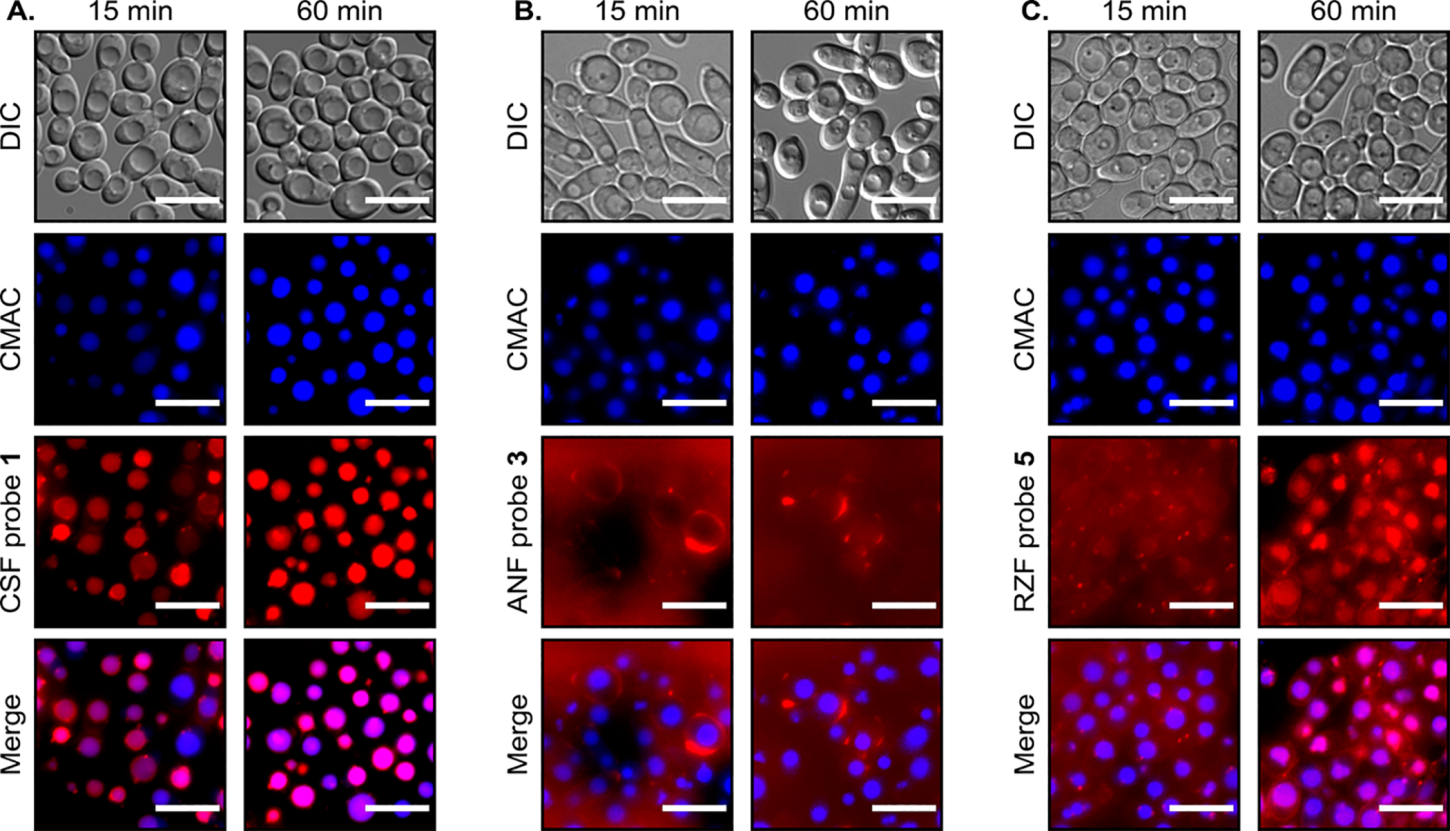
Time-dependent subcellular distribution of TMR-labeled echinocandin probes. Differential interference contrast (DIC) and fluorescent images of *C. albicans* SC5314 yeast cells incubated for 15 or 60 min in PBS with the vacuole-specific fluorescent dye CellTracker Blue CMAC (10 μM, blue) and with (A) CSF probe **1** (1 μM, red), (B) ANF probe **3** (1 μM, red), or (C) RZF probe **5** (1 μM, red). Scale bars, 10 μm. A bandpass filter with an excitation of 560/20 nm and an emission wavelength of 629.5/37.5 nm was used for TMR. A bandpass filter with an excitation wavelength of 350/25 nm and an emission wavelength of 460/25 nm was used for CellTracker Blue CMAC. Each probe was tested in at least in three independent sets of experiments, and at least 1500 cells were captured in each experiment.

The subcellular distribution patterns of the fluorescent echinocandin probes were investigated in cells of two representative echinocandin-susceptible *Candida* strains, *C. albicans* SC5314 and *C. glabrata* strain ATCC 66032, over 60 min of incubation. Comparison of subcellular distributions of TMR-labeled CSF, ANF, and RZF probes (**1**, **3**, and **5**, respectively) and of NBD-labeled CSF, ANF, and RZF probes (**2**, **4**, and **6**, respectively) over time revealed three different and distinguishable patterns (Figures 3 and S9–S11).

Within 15 min after introduction, the CSF probes, TMR-based **1** and NBD-based **2**, localized to the vacuoles of the yeast cells.^32^ Vacuolar accumulation intensified and reached a maximum at approximately 60 min. In contrast, TMR-based and NBD-based ANF probes **3** and **4**, respectively, did not permeate the yeast cells and remained predominantly in the extracellular environment and on the yeast cell surface for the entire 60 min duration of the experiment. The RZF probes **5** and **6** appeared in the extracellular environment and on the yeast cell surface after 15 min; however, after 60 min, both probes localized mainly to the vacuoles. Consistent with this, TMR-labeled probes (**1**, **3**, and **5**) co-localized with the vacuole-specific fluorescent dye CellTracker Blue 7-amino-4-chloromethylcoumarin (CMAC)^41^ after 60 min of incubation in *C. albicans* SC5314, with Pearson correlation values^42^ of 0.73 ± 0.06, 0.03 ± 0.04, and 0.47 ± 0.04, respectively. Similarly, the NBD-labeled echinocandins (**2**, **4**, and **6**) co-localized with CMAC after 60 min of incubation in *C. albicans* SC5314, with Pearson correlation values of 0.40 ± 0.08, 0.15 ± 0.05, and 0.28 ± 0.02, respectively.

If the fluorescent dyes determined the subcellular distribution of the fluorescently labeled echinocandins, all three echinocandins would share the same subcellular localization. However, fluorescently labeled CSF probes accumulated in vacuoles of the yeast cells, fluorescently labeled ANF probes localized predominantly to the extracellular environment and the yeast cell surface, and fluorescently labeled RZF probes appeared in the extracellular environment and on the yeast cell surface shortly after their introduction and were detected in vacuoles after a longer incubation period. Of note, the time-dependent subcellular distribution patterns of the TMR-based echinocandin probes **1**, **3**, and **5** were similar to those of the corresponding NBD-based probes **2**, **4**, and **6** (Figures 3 and S9–S11). The fact that the TMR-labeled and the NBD-labeled echinocandins had similar subcellular distribution patterns, taken together with the large differences in chemical properties of the two dyes, supports the hypothesis that the drug scaffolds, and not the conjugated fluorescent dyes, determined the subcellular distribution patterns of the fluorescent echinocandin probes.

In an attempt to rationalize the distinctively different subcellular distributions of the three echinocandins, our attention was drawn to differences in hydrophobicity/hydrophilicity ratios as evident from their calculated distribution coefficient (log *D*) values, defined as the ratio of concentrations of ionized to neutral species of the compound at the physiological pH of 7.4. CSF probes **1** and **2** were the most hydrophilic of the probes with log*D* values of −7.70 and −6.95, respectively, and ANF probes **3** and **4** were the most hydrophobic with log *D* values of −0.85 and −0.10, respectively. The calculated log *D* values of RZF probes **5** and **6** were −5.08 and −4.32, respectively. The fluorescent probes of the most hydrophobic echinocandin, ANF, accumulated on the cell surface where the target GS complex resides, and those of the most hydrophilic echinocandin, CSF, were cell-permeable. With intermediate log *D* values, RZF probes concentrated on the surface of the yeast cells shortly after introduction and then slowly permeated the cell and accumulated in the vacuoles.

Previously reported mapping of the mutational hotspot regions that confer resistance to echinocandins onto the topology map of Fks suggested that these regions reside near the extracellular membrane surface.^25^ This suggests that echinocandins bind either in close proximity to or directly on the extracellular side of the plasma membrane that houses the echinocandin target Fks. Therefore, echinocandins likely do not need to enter cells to exert their inhibitory effect, and cellular uptake may actually reduce the effective concentration of the drug near Fks.

### Vacuolar Uptake of Fluorescently Labeled CSF is not Prevented by the Presence of the Extracellular-Residing Fluorescently Labeled ANF

**Figure 4:**
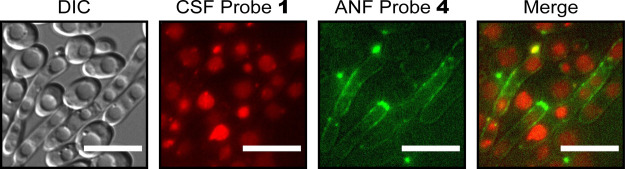
Subcellular distribution of *C. albicans* cells co-incubated with fluorescent probes of two different echinocandins. Differential interference contrast (DIC) and fluorescent images of *C. albicans* SC5314 cells simultaneously incubated with probe **1** (1 μM, red) and with probe **4** (5 μM, green) in PBS for 60 min. Scale bars, 10 μm. A bandpass filter with an excitation of 560/20 nm and an emission wavelength of 629.5/37.5 nm was used for TMR. A bandpass filter with an excitation wavelength of 470/20 nm and an emission wavelength of 525/25 nm was used for NBD.

Fluorescent ANF probes **3** and **4** localize to the yeast cell surface, whereas fluorescent CSF probes **1** and **2** accumulate in vacuoles. The structural similarities between the two echinocandins suggest that the cell surface accumulation of fluorescent ANF can potentially perturb with the uptake of fluorescent CSF into the vacuole. To ask if the intracellular distribution of fluorescently labeled CSF is affected by the presence of fluorescently labeled ANF on the membrane, *C. albicans* SC5314 cells were co-incubated with TMR-labeled CSF probe **1** and with NBD-labeled ANF probe **4** in PBS buffer for 60 min. Since the excitation and emission spectra of NBD and TMR differ considerably, we were able to incubate yeast cells with both fluorescent ANF and CSF and image the two probes in different fluorescent channels. Co-incubation did not alter the subcellular distribution patterns of either of the two fluorescent echinocandins: When co-incubated, probe **1** accumulated in vacuoles, and probe **4** was detected in the extracellular environment and on the surface of the yeast cells (Figure 4). This indicates that the transport mechanism responsible for the internalization of fluorescent CSF is not considerably affected by the extracellular-residing fluorescent ANF.

**Figure 5:**
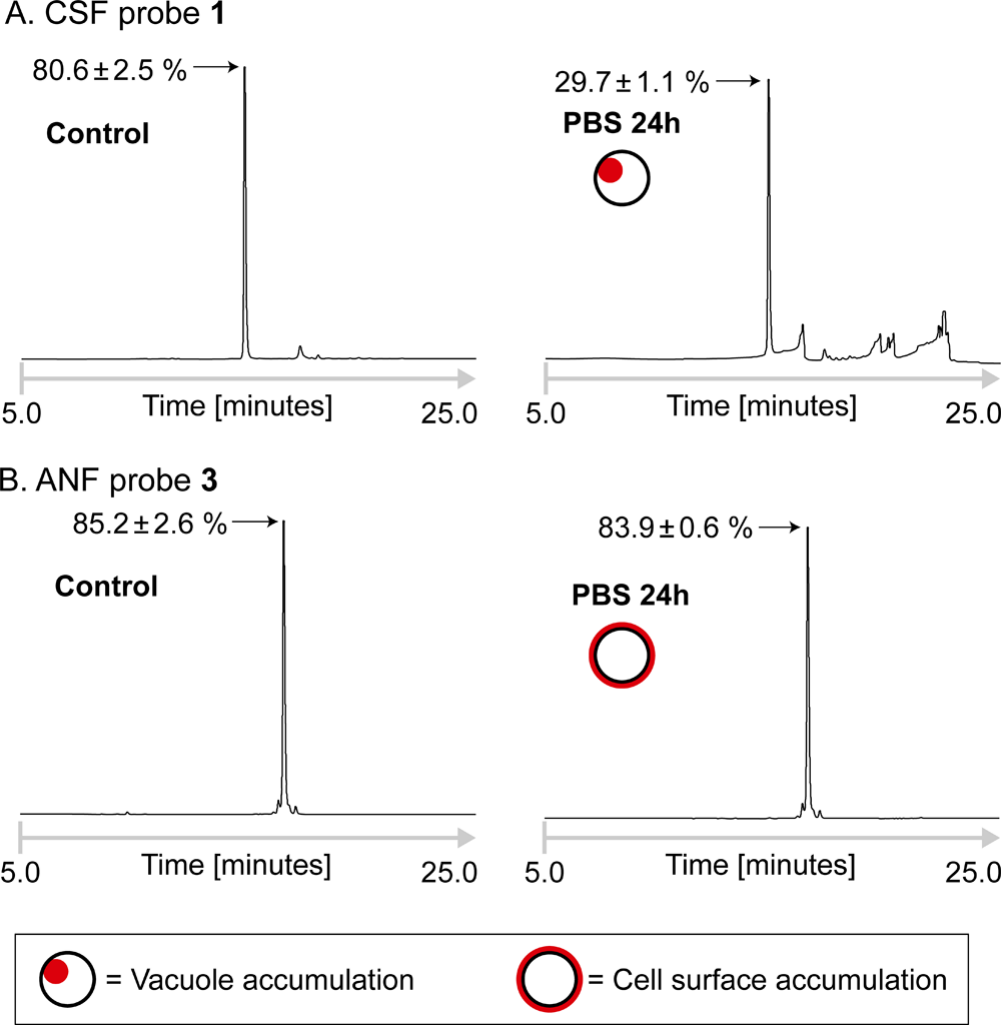
CSF probe **1**, which accumulates in the vacoule of *Candida* yeast cells, is degraded over time, whereas ANF probe **4**, which localizes to the cell surface, remains intact. Reverse-phase HPLC analysis of lysates of *C. albicans* SC5314 yeast cells that were pre-incubated in PBS containing (A) CSF probe **1** or (B) ANF probe **3**. Control: Chromatograms of lysates prepared from yeast cells that were pre-incubated in PBS with the probes for 5 min. PBS 24 h: chromatograms of lysates prepared from yeast cells that were pre-incubated in PBS with the probes for 24 h. The red color indicates the site of probe accumulation during the experiment. The absorbance wavelength was 550 nm. The percentages of intact echinocandin are presented as means of two independent experiments ± standard deviation.

### Vacuolar Uptake Is Associated with Degradation of CSF

The fungal vacuole is an acidic organelle that, similar to the mammalian cell lysosome, is responsible for degradative reactions catalyzed by digestive enzymes and for storage activities. Vacuoles facilitate vesicular trafficking, stress response processes, environmental adaptation, and cell differentiation.^43^ The intra-vacuolar pH is acidic as a result of a vacuolar proton-translocating ATPase that hydrolyzes ATP for transport of protons from the cytosol into the vacuole.^44^ In mammalian cells, many peptide- and protein-based drugs penetrate cells through endocytosis and are trapped in endosomes or degraded by proteases in lysosomes.^44,45^ This prevents these drugs from effectively accomplishing their therapeutic effect. It is therefore reasonable that peptides and proteins that are transported into vacuoles of pathogenic fungi will meet the same fate. Given that echinocandins are lipopeptide drugs and that fluorescently labeled CSF but not ANF accumulated in vacuoles, we speculated that the former might be degraded in the vacuole and, as a result, its efficacy will be limited especially in cases of high-burden infections.

Due to the superior stability and spectral properties of TMR, we used TMR-based CSF and ANF probes **1** and **3**, respectively, for subsequent experiments. These probes were incubated with *C. albicans* SC5314 cells in a nutrient-free PBS buffer. Reverse-phase HPLC analysis of lysates of cells that were pre-incubated with CSF probe **1** revealed that approximately 70% of **1** was degraded after 24 h (Figure 5A). Of note, incubation in the nutrient-rich growth media yeast extract peptone dextrose medium plus adenine (YPAD) resulted in considerably less degradation, approximately 40% after 24 h of incubation (Figure S12). Through a time-dependent investigation of the subcellular distribution of fluorescent CSF, we have previously shown that echinocandins cause cell lysis under conditions that enable rapid yeast cell growth but do not cause lysis under conditions that lead to quiescent cell maintenance.^32^ Thus, growth in the nutrient-rich growth media, which promotes cell lysis and vacuole disassembly, resulted in less degradation of the CSF probe than when the cells were maintained in a nutrient-free buffer. As controls, probes were incubated for a very short period (5 min) with the cells in the PBS or were incubated with the lysate of *C. albicans* SC5314 in the buffer for 24 h (Figures 5A and S13, respectively). In both cases, ∼80% of CSF probe **1** was intact. These results indicate that significant CSF degradation requires prolonged incubation in cells with intact functional vacuoles.

When ANF probe **3** was incubated with cells or lysates of *C. albicans* strain SC5314, no significant decomposition was detected over the 24 h duration of the experiment regardless of the media used (Figures 5B and S12). Given the structural similarities between ANF and CSF, these results may be rationalized by the difference in the subcellular distribution between the two echinocandins: The CSF probe **1** enters the yeast cells via endocytosis^32^ and accumulates in the vacuoles where it is degraded, whereas the ANF probe **3** localizes mainly to the yeast cell surface and remains intact over the course of the experiment.

## Conclusions

To enable exploration of the subcellular distribution of different drugs belonging to the echinocandin class of antifungals, we synthesized fluorescent probes of three drugs in this class; CSF, ANF, and RZF. Imaging of live *Candida* cells incubated with the fluorescent echinocandins provided visible evidence for differences in cell permeability and subcellular distribution of these drugs. CSF probes accumulated in vacuoles of the yeast cells, ANF probes localized predominantly to the extracellular environment and the yeast cell surface, and RZF probes appeared in the extracellular environment and on the yeast cell surface shortly after their introduction and were detected in vacuoles after a longer incubation period. Images of yeast cells that were co-incubated with both fluorescent CSF and ANF probes revealed that vacuolar uptake of fluorescent CSF was not prevented by the presence of the extracellular-residing fluorescent ANF. Comparison of the HPLC chromatograms of extracts of *C. albicans* cells that were pre-incubated with fluorescent CSF or fluorescent ANF revealed extensive degradation of the CSF probe, whereas the cell-surface-residing fluorescent ANF remained intact. This degradation required intact functional vacuoles.

Accumulation of the drug on the fungal cell surface increases its local concentration around the target and may therefore contribute, at least in part, to its efficacy as an inhibitor of the GS complex. Uptake by the vacuole results in decomposition of the echinocandin and, as a consequence, can contribute to a reduction in its efficacy over time. The results of this study therefore suggest that designing echinocandins that localize predominantly to the cell surface of the fungal pathogen should contribute to the metabolic stability and potency of these drugs.

## Materials and Methods

### Preparation of Stock Solutions of the Tested Compounds

The antifungal drug CSF (purchased from S.L. Moran Ltd.), ANF (a gift from Teva Pharmaceutical Industries Ltd.), RZF, and compounds **1**–**6** were dissolved in anhydrous dimethyl sulfoxide (DMSO) to 5 mg/mL or to 1 mM. CellTracker Blue CMAC was purchased from Thermo Fisher and was dissolved in DMSO to 10 mM.

### *Candida* Strains

The laboratory and clinical isolates and ATCC strains used in this study are listed in Table S1. Two *FKS* mutants derived from *C. albicans* SC5314 and two FKS mutants from *C. glabrata* EF1620 were used in this study.

### MIC Broth Double-Dilution Assay

*Candida* strains were streaked from glycerol stock onto YPAD agar plates and grown for 24 h at 30 °C. Colonies were suspended in 1 mL of PBS and diluted to an optical density of 2 × 10^–4^ at 600 nm (OD_600_) in flat-bottom 96-well microplates (Corning) with YPAD broth containing dilutions of each tested compound at concentrations ranging from 64 to 0.008 μg/mL in twofold dilutions. Control wells with yeast cells but no drug and blank wells that contained only YPAD were prepared. MIC values (Table S2) were determined after 24 h at 30 °C by measuring the OD_600_ using a plate reader (Infinite M200 PRO, Tecan). MIC values were defined as the point at which the OD_600_ was reduced by ≥75% compared to the no-drug wells. Each concentration was tested in triplicate, and the results were confirmed by two independent sets of experiments.

### Live Cell Imaging

*Candida* strains were streaked from glycerol stocks onto YPAD agar plates and grown for 24 h at 30 °C. Colonies were grown in tubes in 3 mL of YPAD broth for 24 h at 30 °C with shaking. Cultures were diluted 1:100 and incubated in YPAD broth for 2 h at 30 °C with shaking until log-phase growth was observed. Cultures were then centrifuged, washed with PBS buffer, and resuspended in PBS buffer. Cells were stained with CellTracker Blue CMAC (10 μM) and incubated with TMR-based probes (**1**, **3**, and **5**) at a final concentration of 1 μM or/and with NBD-based probes (**2**, **4**, and **6**) at a final concentration of 5 μM at 30 °C with shaking in the dark over a 2 h time course. To optimize the concentrations of the probes, we investigated the effect of different concentrations (0.1, 1, 5, and 10 μM) of each probe. After washing with PBS, 2 μL aliquots of *Candida* cell samples were placed on glass slides and covered with glass coverslips. Cells were imaged using a Nikon Ti microscope equipped with a Plan Apo VC 100× Oil objective and a Zyla 5.5 sCMOS camera (Andor) using NIS elements Ar software. The bandpass filter sets used to image TMR-based probes (**1**, **3**, and **5**) had an excitation wavelength of 560/20 nm and an emission wavelength of 629.5/37.5 nm. To image NBD-based probes (**2**, **4**, and **6**), the excitation wavelength was 470/20 nm, and the emission wavelength was 525/25 nm. For CellTracker Blue CMAC, an excitation wavelength of 350/25 nm and an emission wavelength of 460/25 nm were used. Images were processed using ImageJ. Each probe was tested in at least three independent sets of experiments, and at least 1500 cells were captured in each experiment. The Pearson correlation values were determined using co-localization analysis with ImageJ.

### Measurement of the Degradation of Echinocandins by *Candida* Cells

*C. albicans* SC5314 cells were streaked from glycerol stocks onto YPAD agar plates and grown for 24 h at 30 °C. Colonies were grown in tubes in 3 mL of YPAD broth for 24 h at 30 °C with shaking. Cultures were diluted 1:50 and incubated in 5 mL of YPAD broth for 3 h at 30 °C with shaking until log-phase growth was observed. Cultures were then centrifuged (4000 rpm, 3 min), washed with PBS buffer, and resuspended in 1 mL of PBS buffer or YPAD. Cells were incubated with TMR-based probes (**1** or **3**) at a final concentration of 10 μM at 30 °C with shaking in the dark for 24 h. After washing three times with PBS, cells were resuspended in 75 μL of TNE buffer (10 mM Tris, 100 mM NaCl, and 1 mM ethylenediaminetetraacetic acid, pH 8.0) in a 1.5 mL Eppendorf tube, and 0.5 mm diameter beads were added to the yeast solution. The mixture was agitated on a vortex at maximum speed for 5 min. Next, 200 μL of 2% Triton X-100 in TNE buffer (v/v) was added to the agitated solution, and then the mixture was sonicated for 15 min. To clarify the yeast lysate, the mixture was centrifuged twice (16 000*g*, 20 min), and the supernatant was removed and filtered using a 0.22 μm syringe filter. The samples were analyzed using analytical reverse-phase HPLC at a 550 nm wavelength. HPLC conditions: mobile phase: acetonitrile in H_2_O (containing 0.1% trifluoroacetic acid (v/v)), gradient from 10 to 90%; flow rate: 1 mL/min; absorbance wavelength: 550 nm. Each sample was tested in two independent sets of experiments. The percentages of intact echinocandin were determined using the area calculation function available in the Hitachi Primaide Software and are presented as means (two replicates) ± standard deviation (SD).

## Supporting Information Available

General chemistry methods and instrumentation; synthetic procedures; ^1^H and ^13^C NMR assignments and high-resolution MS data; analytic HPLC chromatograms; absorption and emission spectra; yeast strains’ information; MIC values; live cell imaging data; HPLC analysis of echinocandins’ degradation; and ^1^H and ^13^C NMR spectra (PDF)

## Abbreviations

CSF: caspofungin
MCF: micafungin
ANF: anidulafungin
RZF: rezafungin
GS: β-glucan synthase
MIC: minimal inhibitory concentration
IC50: half-maximal inhibitory concentrations
TMR: tetramethylrhodamine
NBD: nitrobenzoxadiazole
CMAC: 7-amino-4-chloromethylcoumarin
DIC: differential interference contrast
PBS: phosphate-buffered saline
logD: the distribution coefficient
YPAD: yeast extract peptone dextrose medium plus adenine
SD: standard deviation
DMSO: dimethyl sulfoxide
