# Supplementary material for: Echinocandins Localized to the Target-Harboring Cell Surface Are Not Degraded but Those Entering the Vacuole Are
Source: ACS Chem Biol. 2022 Apr 11;17(5):1155–63. doi: 10.1021/acschembio.2c00060 (PMC9127807; doi:10.1021/acschembio.2c00060)
Supplement: Supplementary file 1 — cb2c00060_si_001.pdf [file cb2c00060_si_001.pdf]

Supporting Information

Echinocandins Localized to the Target-Harboring Cell Surface Are Not Degraded but those Entering the Vacuole Are

*Qais Z. Jaber, Dana Logviniuk, Adi Yona, and Micha Fridman**

School of Chemistry, Raymond & Beverly Sackler Faculty of Exact Sciences, Tel Aviv University, Tel Aviv, 6997801, Israel.

*Correspondence to: Micha Fridman, mfridman@tauex.tau.ac.il.

1. Chemistry

1.1. General chemistry methods and instrumentation.

^{1}H-NMR spectra were recorded on BrukerAvance 400 or 500 MHz spectrometers. ^{13}C-NMR spectra were recorded on BrukerAvance 400 or 500 MHz spectrometers at 100 or 125 MHz. Chemical shifts (reported in ppm) were calibrated to CD_3OD (^{1}H: δ = 3.31, ^{13}C: δ = 49.0). Multiplicities are reported using the following abbreviations: s, singlet; d, doublet; t, triplet; dd, doublet of doublets; ddd, doublet of doublet of doublets; dt, doublet of triplets; m, multiplet. Coupling constants (*J*) are given in Hertz (Hz). High-resolution electrospray ionization mass spectra (HRESI-MS) were measured on a Waters Synapt instrument. Low-resolution electrospray ionization mass spectra (ESI-MS) were measured on a Waters 3100 mass detector. Chemical reactions were monitored by thin-layer chromatography (TLC) (Merck, Silica gel 60 F254). Visualization was achieved using a cerium molybdate stain (5 g $(NH_4)_2Ce(NO_3)_6$, 120 g $(NH_4)_6Mo_7O_{24}\cdot 4H_2O$, 80 mL H_2SO_4, 720 mL H_2O) or with a UV lamp. All chemicals, unless otherwise stated, were obtained from commercial sources. HPLC purification was performed on an ECOM system equipped with a 5-μm, C-18 Phenomenex Luna Axia column (250 mm x 21.2 mm); conditions are given for each compound. Analytical reverse-phase HPLC was performed on a VWR Hitachi instrument equipped with a diode array detector and an Alltech Apollo C18 reverse-phase column (5 μm, 4.6 x 250 mm). The flow rate was 1 mL/min. Solvent A was 0.1% trifluoroacetic acid (TFA) in water (v/v), solvent B was acetonitrile, gradient from 10% to 90%. The SpectraMax i3x Platform spectrophotometer from Molecular Devices was used for fluorescence measurements. log*D* values were calculated using MarvinSketch (version 6.3.1) with default parameters and with an electrolyte concentration of 0.1 M NaCl at physiological pH (7.4).

1.2. Synthetic procedures.

Compound 2a. Anidulafungin (ANF) (100 mg, 0.087 mmol, 1 eq.) dissolved in dry dimethyl sulfoxide (DMSO) (4 mL) was treated with 3-butyn-1-ol (140.2 mg, 152 μL, 2 mmol, 25 eq.) and with HCl (4 M in

1,4-dioxane, 20 μL, 1 eq.). The solution was stirred at ambient temperature for 2 days, and the disappearance of ANF ($[M-H]^-$ m/z 1138) and the appearance of **2a** ($[M-H]^-$ m/z 1190) were monitored by ESI-MS. Upon completion of the reaction, the solution was diluted with acetonitrile:H_2O. Purification by preparative RP-HPLC (mobile phase: acetonitrile in H_2O containing 0.1% TFA (v/v); gradient from 10% to 90%; flow rate: 15 mL/min) yielded compound **2a** (65 mg, 62%) as a white powder. HRESI-MS m/z calculated for $C_{62}H_{77}N_7O_{17}Na$,1214.5274; found $[M+Na]^+$, 1214.5262. ^1H NMR (400 MHz, CD_3OD) δ (ppm) 7.97 (d, *J* = 8.4 Hz, 2H), 7.68–7.80 (m, 6H), 7.62 (d, *J* = 9.0 Hz, 2H), 7.18 (d, *J* = 9.0 Hz, 2H), 7.02 (d, *J* = 8.4 Hz, 2H), 6.79 (d, *J* = 9.0 Hz, 2H), 5.27 (d, *J* = 2.8 Hz, 1H), 5.03 (d, *J* = 3.2 Hz, 1H), 4.92 (d, *J* = 4.3 Hz, 1H), 4.73 (dd, *J* = 11.7, 5.7 Hz, 1H), 4.64 (dd, *J* = 11.3, 7.2 Hz, 1H), 4.53–4.60 (m, 2H), 4.43 (d, *J* = 2.8 Hz, 1H), 4.34 (d, *J* = 7.8 Hz, 2H), 4.20–4.30 (m, 3H), 4.09–4.16 (m, 3H), 4.04 (t, *J* = 6.5 Hz, 2H), 3.99 (dd, *J* = 11.1, 2.8 Hz, 1H), 3.91 (dd, *J* = 9.5, 7.3 Hz, 1H), 3.84 (d, *J* = 11.0 Hz, 1H), 3.58–3.74 (m, 2H), 3.44 (t, *J* = 9.1 Hz, 1H), 2.51–2.59 (m, 1H), 2.41–2.50 (m, 3H), 2.23–2.32 (m, 1H), 2.19 (t, *J* =2.7 Hz, 1H), 1.76–1.87 (m, 2H), 1.39–1.56 (m, 4H), 1.24–1.35 (m, 6H), 1.09 (d, *J* = 6.9 Hz, 3H), 0.99 (t, *J* = 7.2 Hz, 3H).^{13}C NMR (125 MHz, CD_3OD) δ (ppm) 174.3, 174.2, 173.6, 172.8, 172.6, 170.1, 169.8, 160.4, 158.5, 145.3, 141.9, 139.3, 133.9, 133.8, 133.1, 129.6, 129.3, 128.9, 128.5, 128.0, 127.7, 116.2, 115.9, 82.0, 81.1, 77.0, 75.8, 71.3, 70.6, 70.4, 69.7, 69.5, 69.1, 68.3, 67.5, 62.5, 58.9, 58.8, 57.2, 57.0, 56.3, 53.0, 52.2, 39.2, 38.6, 35.2, 30.2, 29.4, 23.6, 20.3, 20.1, 19.7, 14.4, 11.3.

Compound 3a. RZF as a chloride salt (119 mg, 0.095 mmol, 1 eq.) was dissolved in dry *N*,*N*-dimethylformamide (DMF) (5 mL), treated with cesium carbonate (87 mg, 0.28 mmol, 3 eq.), and stirred for 10 minutes at ambient temperature. Then 80% propargyl bromide solution in toluene (w/v) (15.5 μL, 0.139 mmol, 1.5 eq.) was added. The reaction mixture was stirred at ambient temperature for 16 h, and progress was monitored following the disappearance of RZF ($[M]^+$ m/z 1225) and the appearance of **3a** ($[M]^+$ m/z 1263) by ESI-MS. Upon completion of the reaction, the solution was diluted with acetonitrile:H_2O. Purification by preparative RP-HPLC (mobile phase: acetonitrile in H_2O containing 0.1% TFA (v/v); gradient from 10% to 90%; flow rate: 15 mL/min) yielded compound **3a** (60 mg, 49%) as a

white powder. HRESI-MS m/z calculated for $C_{66}H_{87}N_8O_{17}$,1263.6189; found $[M]^+$, 1263.6198. ^{1}H NMR (500 MHz, CD_3OD) δ (ppm) 7.97 (d, *J* = 8.4 Hz, 2H), 7.79 (d, *J* = 8.4 Hz, 2H), 7.74 (d, *J* = 8.5 Hz, 2H), 7.70 (d, *J* = 8.5 Hz, 2H), 7.60 (d, *J* = 8.7 Hz, 2H), 7.27 (d, *J* = 8.7 Hz, 2H), 6.95–7.04 (m, 4H), 5.43 (d, *J* = 2.1 Hz, 1H), 5.04 (d, *J* = 3.2 Hz, 1H), 4.88–4.92 (m, 1H), 4.75 (dd, *J* = 12.1, 5.0 Hz, 1H), 4.72 (d, *J* = 2.4 Hz, 2H), 4.54–4.61 (m, 3H), 4.35–4.41 (m, 3H), 4.23–4.27 (m, 2H), 4.14–4.20 (m, 1H), 3.87–4.11 (m, 7H), 3.82 (d, *J* = 10.7 Hz, 1H), 3.62 (ddd, *J* = 13.8, 7.2, 2.1 Hz, 1H), 3.53 (ddd, *J* = 13.9, 6.2, 2.2 Hz, 1H), 3.47 (dd, *J* = 9.8, 7.0 Hz, 1H), 3.14 (s, 9H), 2.93 (t, *J* = 2.4 Hz, 1H), 2.51 (dt, *J* = 12.2, 6.8 Hz, 1H), 2.44 (dd, *J* = 13.0, 7.0 Hz, 1H), 2.28 (ddd, *J* = 13.9, 9.3, 5.0 Hz, 1H), 2.01–2.11 (m, 2H), 1.76–1.85 (m, 2H), 1.38–1.53 (m, 4H), 1.22–1.29 (m, 6H), 1.07 (d, *J* = 6.9 Hz, 3H), 0.96 (t, *J* = 7.2 Hz, 3H). ^{13}C NMR (100 MHz, CD_3OD) δ (ppm) 174.3, 173.8, 173.5, 172.8, 172.6, 170.1, 169.5, 160.5, 159.0, 145.6, 142.0, 139.1, 135.3, 133.9, 133.7, 129.5, 129.2, 128.9, 128.5, 128.1, 127.9, 116.0, 115.9, 81.5, 79.8, 76.9, 76.8, 76.2, 75.6, 71.3, 70.0, 69.8, 69.1, 68.3, 68.2, 66.7, 62.7, 62.6, 58.8, 57.2, 57.0, 56.6, 56.2, 54.7, 53.3, 51.9, 39.0, 38.6, 36.2, 30.2, 29.4, 23.6, 20.0, 19.7, 14.4, 11.6.

Synthesis of probe 1. Probe **1** was prepared as previously reported[1] with minor changes. Compound **1a** (35 mg, 0.024 mmol, 1 eq.) and azide-functionalized TMR (25 mg, 0.048 mmol, 2 eq.) were dissolved in DMF (3 mL). A catalytic amount of $CuSO_4 \cdot 5H_2O$ and sodium ascorbate were added to the solution. The reaction solution was stirred at ambient temperature for 2.5 h, and the disappearance of **1a** ($[M-H]^-$ m/z 1430.6) and the appearance of Boc-protected probe **1** ($[M-H]^-$ m/z 1942.6) were monitored by ESI-MS. Upon completion of the reaction, solvent was removed by lyophilization. The crude powder was then dissolved in isopropanol (4 mL), and 32% HCl (w/v) (2 mL) was slowly added dropwise. The reaction was stirred at ambient temperature for 2 h. Progress was monitored by following the disappearance of Boc-protected probe **1** ($[M-H]^-$ m/z 1942.6) and the appearance of probe **1** ($[M-H]^-$ m/z 1642.6) using ESI-MS. Upon completion of the reaction, the solution was diluted with acetonitrile:H_2O, and preparative reverse-phase HPLC (mobile phase: acetonitrile in H_2O containing 0.1% TFA (v/v); gradient from 10% to 90%; flow rate: 15 mL/min) was used to purify the hydrochloride salt of probe **1** (40 mg, 95%) as a red powder.

Synthesis of probe 2. Probe **2** was prepared as previously reported[1] with minor changes. Compound **1a** (50 mg, 0.035 mmol, 1 eq.) and azide-functionalized NBD (18.4 mg, 0.07 mmol, 2 eq.) were dissolved in dry DMF (4 mL). A catalytic amount of $CuSO_4 \cdot 5H_2O$ and sodium ascorbate were added to the solution. The reaction solution was stirred at ambient temperature for 11 h, and progress was monitored by following the disappearance of **1a** ($[M-H]^-$ m/z 1430.6) and the appearance of Boc-protected probe **2** ($[M-H]^-$ m/z 1693.3) using ESI-MS. Upon completion of the reaction, the solvent was removed by lyophilization. The crude powder was then dissolved in isopropanol (8 mL), and 32% HCl (w/v) (4 mL) was slowly added dropwise. The reaction was stirred at ambient temperature for 2 h. Progress was monitored by following the disappearance of Boc-protected probe **2** ($[M-H]^-$ m/z 1693.3) and the appearance of probe **2** ($[M-H]^-$ m/z 1393.1) using ESI-MS. Upon completion of the reaction, the solution was diluted with acetonitrile:H_2O, and preparative reverse-phase HPLC (mobile phase: acetonitrile in H_2O containing 0.1% TFA (v/v); gradient from 10% to 90%; flow rate: 15 mL/min) was used to purify the hydrochloride salt of probe **2** (26.5 mg, 52%) as an orange powder.

Synthesis of probe 3. Compound **2a** (26 mg, 0.022 mmol, 1 eq.) and azide-functionalized TMR (17 mg, 0.033 mmol, 1.5 eq.) were dissolved in dry DMF (2 mL). A catalytic amount of $CuSO_4 \cdot 5H_2O$ and sodium ascorbate were added to the solution. The reaction solution was stirred at ambient temperature overnight, and progress was monitored by following the disappearance of **2a** ($[M+Na]^+$ m/z 1214) and the appearance of **3** ($[M+H]^+$ m/z 1705) using ESI-MS. Upon completion of the reaction, the solution was diluted with acetonitrile:H_2O. Preparative reverse-phase HPLC (mobile phase: acetonitrile in H_2O containing 0.1% TFA (v/v); gradient from 10% to 90%; flow rate: 15 mL/min) yielded probe **3** (27 mg, 73%) as a red powder. HRESI-MS m/z calculated for $C_{90}H_{105}N_{13}O_{21}Na$, 1726.7446; found $[M+Na]^+$, 1726.7456. ^1H NMR (500 MHz, CD_3OD) δ (ppm) 8.62 (d, *J* = 1.8 Hz, 1H), 8.19 (dd, *J* = 7.9, 1.8 Hz, 1H), 7.83 (d, *J* = 6.8 Hz, 3H), 7.61–7.68 (m, 6H), 7.56 (d, *J* = 8.6 Hz, 2H), 7.34 (d, *J* = 7.9 Hz, 1H), 7.12 (d, *J* = 8.6 Hz, 2H), 6.91–7.05 (m, 6H), 6.83 (dd, *J* = 14.1, 2.3 Hz, 2H), 6.75 (d, *J* = 8.3 Hz, 2H), 5.22 (d, *J* = 2.8 Hz, 1H), 4.97 (d, *J* = 3.1 Hz, 1H), 4.89–4.92 (m, 1H), 4.66 (dd, *J* = 11.3, 6.0 Hz, 1H), 4.42–4.57 (m,

6H), 4.40 (d, *J* = 3.2 Hz, 1H), 4.29 (d, *J* = 8.1 Hz, 1H), 4.17–4.24 (m, 3H), 4.05–4.11 (m, 1H), 4.00 (t, *J* = 6.5 Hz, 2H), 3.94 (dd, *J* = 11.1, 3.1 Hz, 1H), 3.86 (dd, *J* = 9.5, 7.3 Hz, 1H), 3.76–3.82 (m, 2H), 3.66–3.73 (m, 1H), 3.45–3.52 (m, 2H), 3.40 (t, *J* = 8.8 Hz, 1H), 3.22–3.25 (m, 12H), 2.84–2.91 (m, 2H), 2.44–2.52 (m, 1H), 2.37 (dd, *J* = 13.0, 7.1 Hz, 1H), 2.21–2.30 (m, 3H), 1.95–2.07 (m, 2H), 1.76–1.83 (m, 2H), 1.38–1.52 (m, 4H), 1.22–1.28 (m, 6H), 1.03 (d, *J* = 6.9 Hz, 3H), 0.99 (t, *J* = 7.2 Hz, 3H).^{13}C NMR (125 MHz, CD_3OD) δ (ppm) 174.3, 174.2, 173.6, 173.3, 172.7, 172.5, 170.2, 169.5, 168.2, 167.5, 160.5, 160.4, 159.0, 158.9, 158.8, 158.5, 146.5, 145.0, 141.7, 139.0, 138.1, 137.3, 133.7, 133.1, 132.8, 132.6, 132.0, 131.8, 131.0, 129.6, 129.2, 128.9, 128.4, 127.9, 127.6, 124.6, 116.2, 116.0, 115.6, 114.8, 114.7, 97.4, 81.2, 77.0, 75.7, 71.3, 70.3, 69.8, 69.1, 69.0, 68.2, 67.5, 62.4, 58.8, 58.7, 57.2, 57.1, 56.2, 53.1, 52.4, 41.0, 39.1, 38.8, 36.6, 35.2, 30.8, 30.5, 30.2, 29.4, 27.1, 23.6, 20.1, 19.8, 14.4, 11.4.

Synthesis of probe 4. Compound **2a** (25 mg, 0.021 mmol, 1 eq.) and azide-functionalized NBD (11 mg, 0.042 mmol, 2 eq.) were dissolved in dry DMF (2 mL). A catalytic amount of $CuSO_4 \cdot 5H_2O$ and sodium ascorbate were added to the solution. The reaction solution was stirred at ambient temperature for 15 h, and progress was monitored by following the disappearance of **2a** ($[M+Na]^+$ m/z 1214) and the appearance of **4** ($[M+Na]^+$ m/z 1478) using ESI-MS. Upon completion of the reaction, the solution was diluted with acetonitrile:H_2O. Purification by preparative reverse-phase HPLC (mobile phase: acetonitrile in H_2O containing 0.1% TFA (v/v); gradient from 10% to 70%; flow rate: 15 mL/min) yielded probe **4** (16.7 mg, 55%) as an orange powder. HRESI-MS m/z calculated for $C_{71}H_{86}N_{14}O_{20}Na$,1477.6041; found $[M+Na]^+$, 1477.6019. ^{1}H NMR (400 MHz, CD_3OD) δ (ppm) 8.35 (d, *J* = 9.0 Hz, 1H), 7.90 (d, *J* = 8.4 Hz, 2H), 7.72 (d, *J* = 8.4 Hz, 2H), 7.57–7.70 (m, 5H), 7.53 (d, *J* = 8.8 Hz, 2H), 7.15 (d, *J* = 8.6 Hz, 2H), 6.96 (d, *J* = 8.8 Hz, 2H), 6.76 (d, *J* = 8.2 Hz, 2H), 6.11 (d, *J* = 8.9 Hz, 1H), 5.23 (d, *J* = 2.8 Hz, 1H), 5.00 (d, *J* = 2.9 Hz, 1H), 4.87–4.91 (m, 1H), 4.71 (dd, *J* = 10.9, 5.9 Hz, 1H), 4.63 (dd, *J* = 10.9, 7.3 Hz, 1H), 4.51–4.58 (m, 2H), 4.30–4.42 (m, 5H), 4.27 (dd, *J* = 7.8, 1.6 Hz, 1H), 4.18–4.24 (m, 2H), 4.00–4.05 (m, 3H), 3.97 (dd, *J* = 11.1, 3.4 Hz, 1H), 3.79–3.90 (m, 3H), 3.71–3.77 (m, 1H), 3.36–3.45 (m, 3H), 2.83–3.00 (m, 2H), 2.42–2.54 (m, 2H), 2.16–2.28 (m, 3H), 1.95–2.12 (m, 2H), 1.75–1.84 (m, 2H), 1.38–1.54 (m, 4H),

1.22–1.31 (m, 6H), 1.05 (d, *J* = 6.9 Hz, 3H), 0.97 (t, *J* = 7.1 Hz, 3H). ^{13}C NMR (125 MHz, CD_3OD) δ (ppm) 172.7, 172.1, 171.4, 171.1, 168.9, 168.0, 159.1, 157.1, 145.3, 143.7, 140.4, 137.5, 136.9, 132.4, 132.2, 131.2, 128.2, 127.8, 127.4, 126.9, 126.5, 126.3, 122.8, 114.8, 114.5, 79.8, 75.5, 74.4, 74.2, 69.9, 68.8, 68.3, 67.9, 67.7, 66.8, 66.4, 61.1, 57.3, 55.8, 55.7, 54.9, 51.6, 50.8, 37.7, 37.3, 34.0, 29.4, 28.8, 28.0, 25.4, 22.2, 18.7, 18.3, 13.0, 9.9.

Synthesis of probe 5. Compound **3a** (28.2 mg, 0.022 mmol, 1 eq.) and azide-functionalized TMR (17.2 mg, 0.033 mmol, 1.5 eq.) were dissolved in dry DMF (2 mL). A catalytic amount of $CuSO_4 \cdot 5H_2O$ and sodium ascorbate were added to the solution. The solution was stirred at ambient temperature overnight, and progress was monitored by following the disappearance of **3a** ($[M]^+$ m/z 1263) and the appearance of **5** ($[M]^+$ m/z 1776) using ESI-MS. Upon completion of the reaction, the solution was diluted with acetonitrile:H_2O. Purification by preparative reverse-phase HPLC (mobile phase: acetonitrile in H_2O containing 0.1% TFA (v/v); gradient from 10% to 90%; flow rate: 15 mL/min) yielded probe **5** (27 mg, 68%) as a red powder. HRESI-MS m/z calculated for $C_{94}H_{115}N_{14}O_{21}$, 1775.8361; found $[M]^+$, 1775.8373. ^1H NMR (500 MHz, CD_3OD) δ (ppm) 8.70 (d, *J* = 1.7 Hz, 1H), 8.18 (dd, *J* = 7.9, 1.7 Hz, 1H), 8.13 (s, 1H), 7.96 (d, *J* = 8.4 Hz, 2H), 7.79 (d, *J* = 8.3 Hz, 2H), 7.73 (d, *J* = 8.5 Hz, 2H), 7.69 (d, *J* = 8.5 Hz, 2H), 7.60 (d, *J* = 8.8 Hz, 2H), 7.46 (d, *J* = 7.9 Hz, 1H), 7.25 (d, *J* = 8.7 Hz, 2H), 7.15 (dd, *J* = 9.5, 0.8 Hz, 2H), 6.93–7.05 (m, 8H), 5.43 (s, 1H), 5.16 (s, 2H), 5.03 (s, 1H), 4.88–4.91 (m, 1H), 4.74 (dd, *J* = 11.8, 5.3 Hz, 1H), 4.51–4.61 (m, 5H), 4.30–4.42 (m, 3H), 4.22–4.27 (m, 2H), 4.14–4.20 (m, 1H), 3.86–4.11 (m, 7H), 3.81 (d, *J* = 10.9 Hz, 1H), 3.62 (ddd, *J* = 13.5, 7.3, 1.9 Hz, 1H), 3.42–3.56 (m, 4H), 3.29 (s, 12H), 3.14 (s, 9H), 2.50 (dt, *J* = 13.5, 6.8 Hz, 1H), 2.43 (dd, *J* = 12.8, 7.0 Hz, 1H), 2.23–2.35 (m, 3H), 1.99–2.11 (m, 2H), 1.77–1.84 (m, 2H), 1.38–1.53 (m, 4H), 1.21–1.28 (m, 6H), 1.07 (d, *J* = 6.9 Hz, 3H), 0.97 (t, *J* = 7.2 Hz, 3H). ^{13}C NMR (100 MHz, CD_3OD) δ (ppm) 174.2, 173.8, 173.4, 172.7, 172.6, 170.1, 169.5, 168.3, 167.4, 160.6, 160.5, 159.6, 159.0, 158.9, 145.5, 145.0, 142.0, 139.1, 138.2, 137.5, 135.2, 133.8, 133.7, 132.9, 132.3, 131.9, 131.3, 129.7, 129.2, 128.9, 128.5, 128.0, 127.9, 127.8, 127.7, 125.6, 116.0, 115.6, 114.7,

97.5, 81.4, 77.0, 76.2, 75.6, 71.3, 69.9, 69.8, 69.1, 68.3, 68.2, 66.7, 62.7, 62.6, 62.5, 58.8, 57.2, 57.0, 56.2, 54.7, 53.3, 51.9, 41.0, 39.0, 38.6, 36.2, 30.9, 30.7, 30.2, 29.4, 23.6, 20.0, 19.7, 14.4, 11.6.

Synthesis of compound 6. Compound **3a** (25 mg, 0.0196 mmol, 1 eq.) and azide-functionalized NBD (10.4 mg, 0.0391 mmol, 2 eq.) were dissolved in dry DMF (2 mL). A catalytic amount of $CuSO_4{\cdot}5H_2O$ and sodium ascorbate were added to the solution. The reaction solution was stirred at ambient temperature overnight, and progress was monitored by following the disappearance of **3a** ($[M]^+$ m/z 1263) and the appearance of **6** ($[M]^+$ m/z 1527) using ESI-MS. Upon completion of the reaction, the solution was diluted with acetonitrile:H_2O. Purification by preparative reverse-phase HPLC (mobile phase: acetonitrile in H_2O containing 0.1% TFA (v/v); gradient from 10% to 90%; flow rate: 15 mL/min) yielded probe **6** (11 mg, 37%) as an orange powder. HRESI-MS m/z calculated for $C_{75}H_{96}N_{15}O_{20}$, 1526.6956; found $[M]^+$, 1526.6965. ^1H NMR (400 MHz, CD_3OD) δ (ppm) 8.51 (d, *J* = 8.8 Hz, 2H), 8.06 (s, 2H), 7.97 (d, *J* = 8.4 Hz, 2H), 7.80 (d, *J* = 8.5 Hz, 2H), 7.67–7.77 (m, 4H), 7.60 (d, *J* = 8.7 Hz, 2H), 7.25 (d, *J* = 8.4, 2H), 6.96–7.05 (m, 4H), 6.27 (d, *J* = 9.0 Hz, 1H), 5.42 (d, *J* = 2.1 Hz, 1H), 5.15 (s, 2H), 5.04 (d, *J* = 3.0 Hz, 1H), 4.88–4.91 (m, 1H), 4.73–4.77 (m, 1H), 4.54–4.63 (m, 5H), 4.36 (dd, *J* = 6.2, 1.7 Hz, 2H), 4.29 (d, *J* = 1.5 Hz, 1H), 4.22–4.27 (m, 2H), 4.14–4.19 (m, 1H), 3.86–4.09 (m, 7H), 3.82 (d, *J* = 10.9 Hz, 1H), 3.43–3.65 (m, 5H), 3.14 (s, 9H), 2.24–2.52 (m, 5H), 1.99–2.12 (m, 2H), 1.77–1.84 (m, 2H), 1.39–1.52 (m, 4H), 1.22–1.28 (m, 6H), 1.05 (d, *J* = 7.1 Hz, 3H), 0.91 (t, *J* = 7.1 Hz, 3H). ^{13}C NMR (125 MHz, CD_3OD) δ (ppm) 172.8, 172.3, 172.1, 171.3, 171.2, 168.8, 168.1, 159.1, 158.1, 144.2, 143.8, 140.6, 137.7, 137.3, 137.1, 133.8, 132.5, 132.3, 128.3, 127.7, 127.5, 127.1, 126.6, 126.5, 124.2, 114.6, 114.5, 80.1, 75.6, 74.7, 74.2, 69.9, 68.6, 68.4, 67.7, 66.9, 66.8, 65.3, 61.3, 61.1, 61.0, 57.2, 55.8, 55.6, 54.8, 53.3, 51.9, 50.5, 40.5, 37.6, 37.2, 34.8, 31.7, 29.3, 28.8, 28.0, 22.2, 18.5, 18.3, 13.0, 10.1.

1.3. Analytical HPLC chromatograms

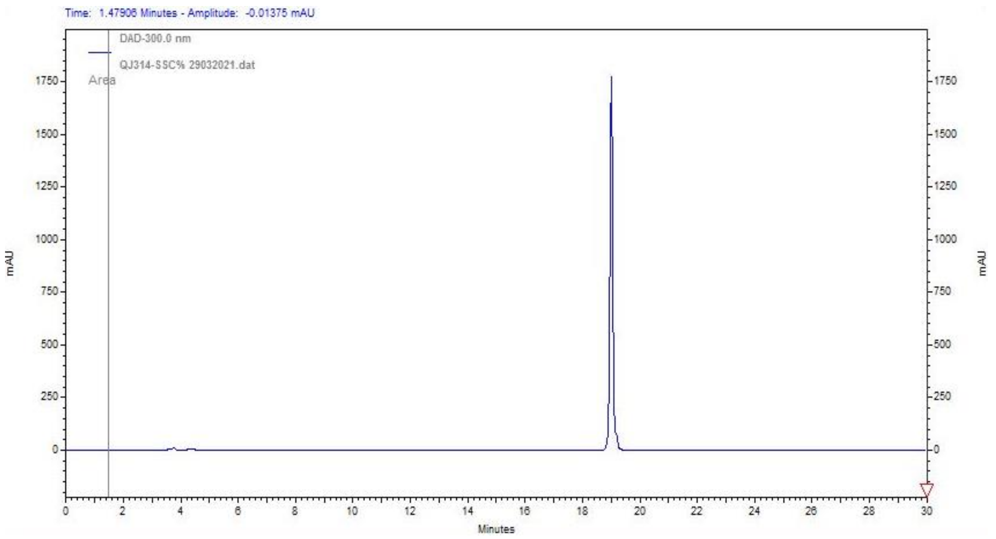

Figure S1. Analytical RP-HPLC chromatogram (diode array detector) of compound **2a**.

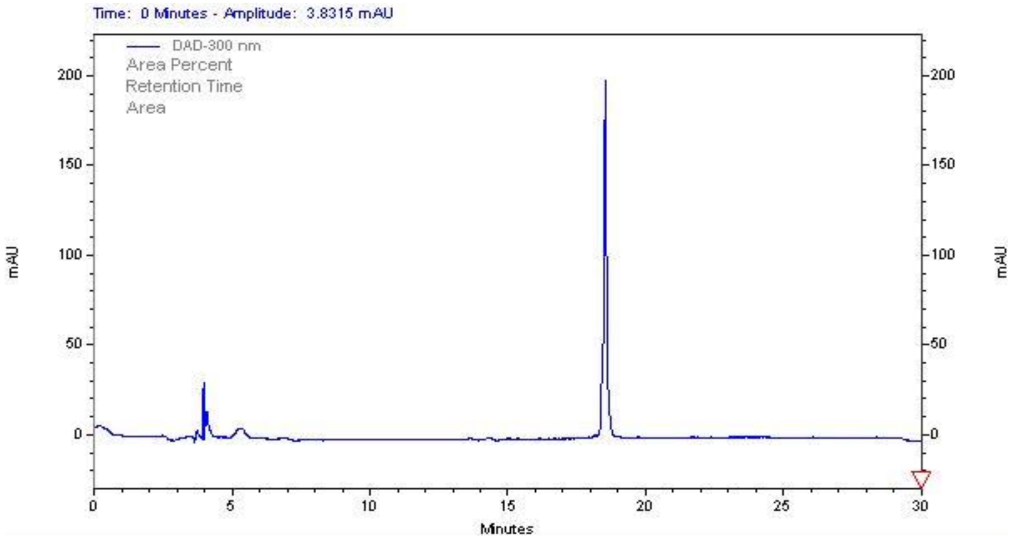

Figure S2. Analytical RP-HPLC chromatogram (diode array detector) of compound **3a**.

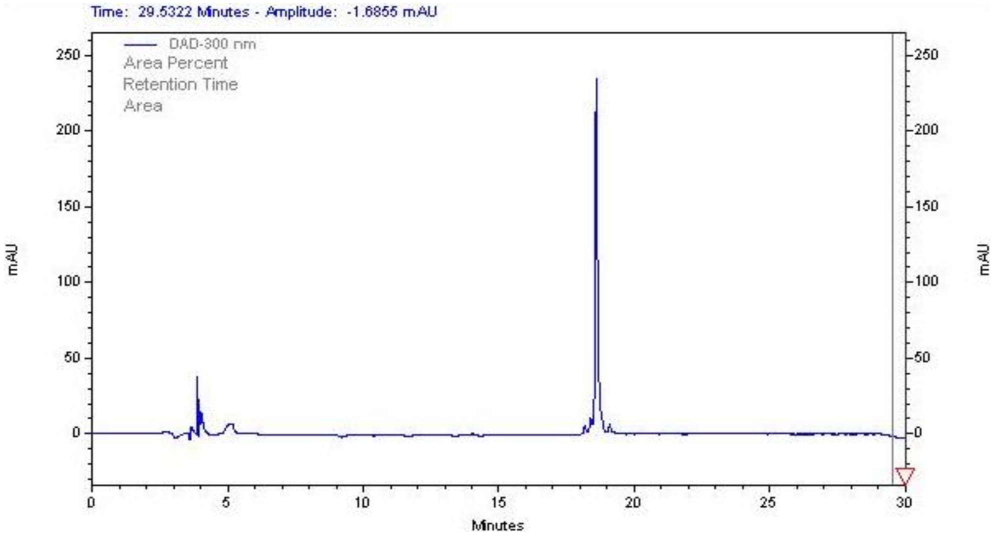

Figure S3. Analytical RP-HPLC chromatogram (diode array detector) of compound **3**.

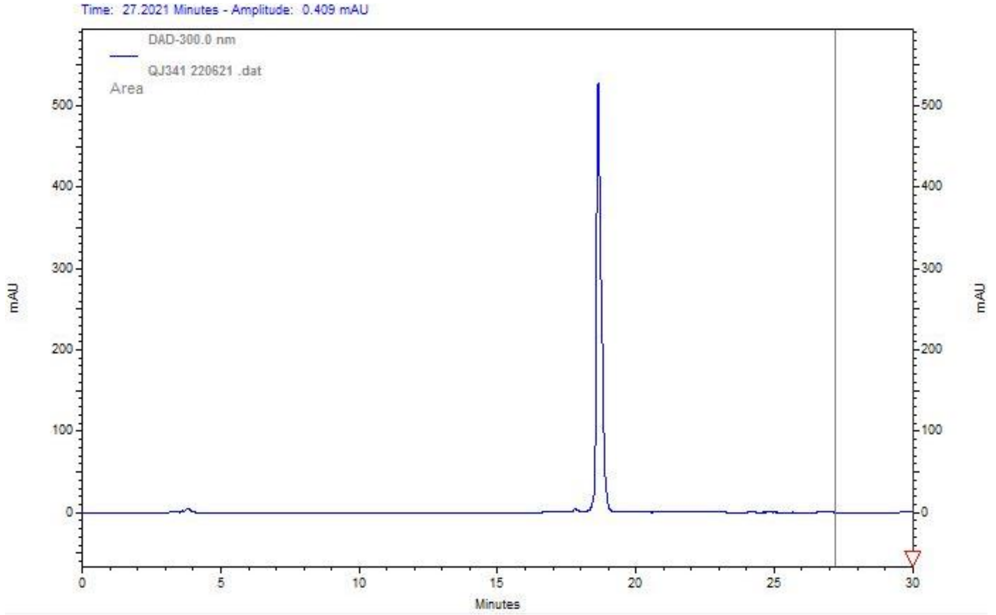

Figure S4. Analytical RP-HPLC chromatogram (diode array detector) of compound **4**.

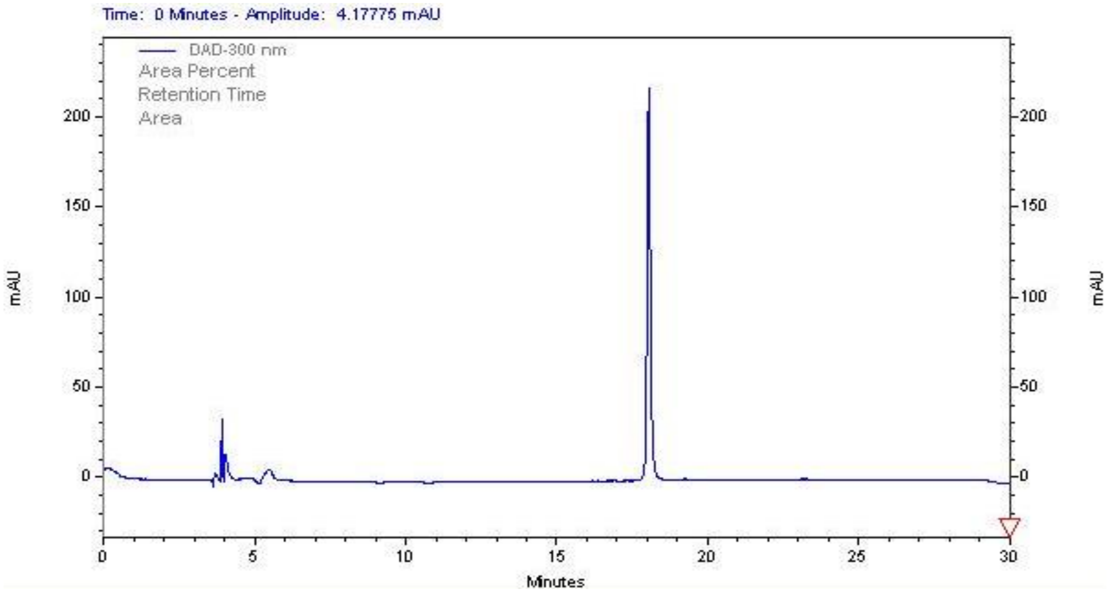

Figure S5. Analytical RP-HPLC chromatogram (diode array detector) of compound **5**.

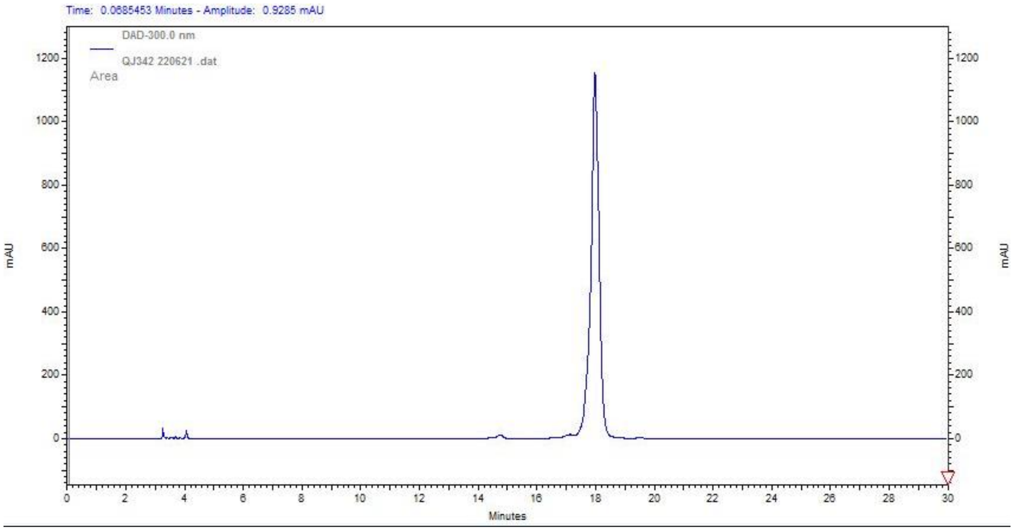

Figure S6. Analytical RP-HPLC chromatogram (diode array detector) of compound **6**.

1.4. Absorption / emission spectra

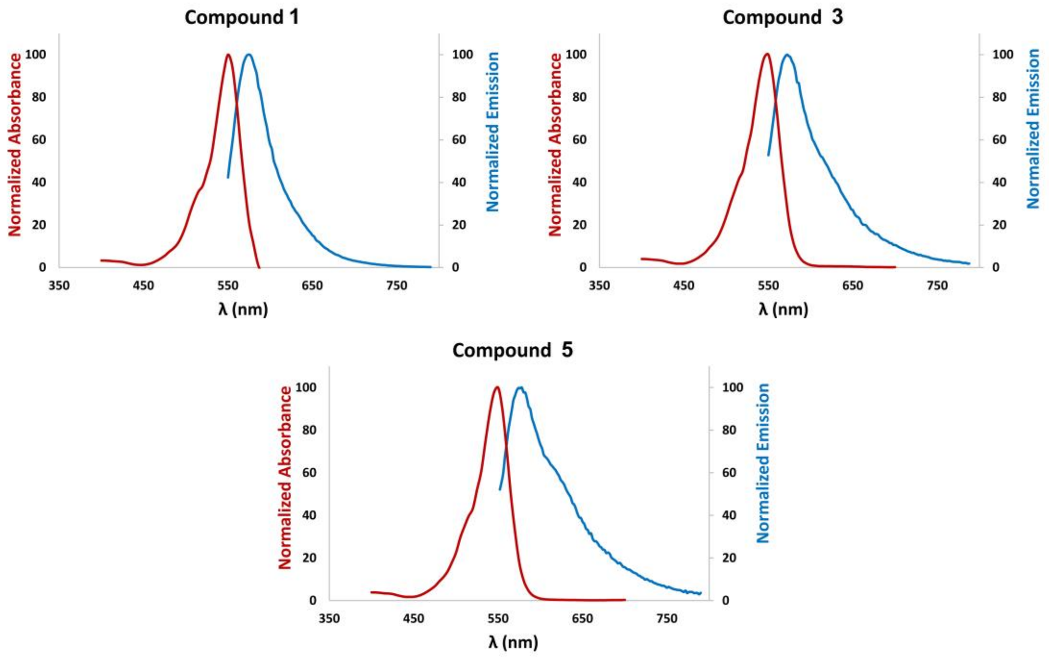

Figure S7. Normalized absorption and emission spectra of TMR-labeled echinocandin probes **1**, **3**, and **5**. The measurements were made at the concentration of 10 μM in EtOH.

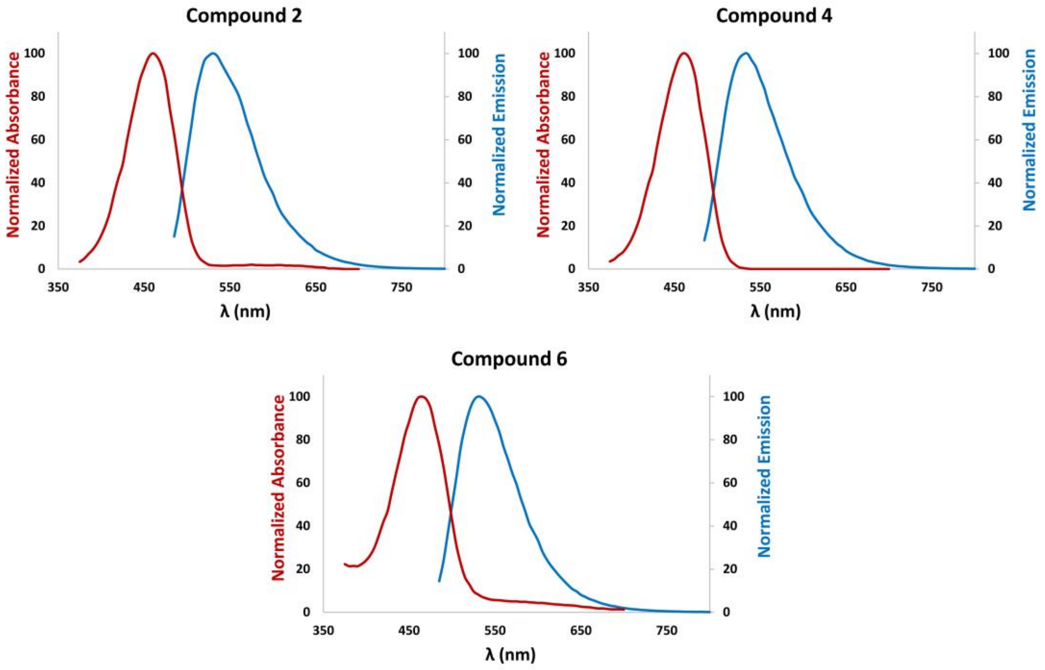

Figure S8. Normalized absorption and emission spectra of NBD-labeled echinocandin probes **2**, **4**, and **6**. The measurements were made at the concentration of 10 μM in EtOH.

2. Biology

2.1. *Candida* strains.

Table S1. Strains information.

#	Species	Strain name	Isogenic parental strain	Genotype	Source
**A.**	*C. albicans*	SC5314	WT		David Perlin[3]
**B.**	*C. albicans*	SN152	–		Susan Lindquist[4]
**C.**	*C. albicans*	ATCC 24433	–		ATCC
**D.**	*C. albicans*	ATCC 10231	–		Susan Lindquist
**E.**	*C. albicans*	ATCC 90028	–		ATCC
**F.**	*C. albicans*	DPL1016 (DP-A15-10)	SC5314	*FKS1* mutant HS1-S645P	David Perlin[5]
**G.**	*C. albicans*	T-2068 (DP-C42)	SC5314	*FKS1* HS1-F641S mutation	David Perlin[5]
**H.**	*C. glabrata*	EF1620			Toni Gabaldon[6]
**I.**	*C. glabrata*	ATCC 2001	–		Cecile Fairhead[7]
**J.**	*C. glabrata*	ATCC 66032	–		ATCC
**K.**	*C. glabrata*	ATCC 90030	WT		David Perlin
**L.**	*C. glabrata*	T-192 (1775)	clinical isolate		Ronen Ben Ami[8]
**M.**	*C. glabrata*	TGL00275	EF1620	*FKS2* HS1-L662F-D666N	Toni Gabaldon[1]
**N.**	*C. glabrata*	TGL00277	EF1620	*FKS1* HS1-F625Y-D632E	Toni Gabaldon[1]

2.2. Minimal inhibitory concentration.

Table S2. Antifungal activities (MICs) of parent (**CSF**, **ANF**, and **RZF**) and fluorescent echinocandins (TMR-based probes **1**, **3**, **5** and the corresponding NBD-based probes **2**, **4**, **6**).[a]

#	Strain name	Compound MIC [μg/mL]									
		**CSF**	**ANF**	**RZF**	**1**	**2**	**3**	**4**	**5**	**6**	**Free dyes**[b]
**A.**	SC5314	0.031	0.008	0.016	0.5	1	1	0.25	2	0.5	>64
**B.**	SN152	0.031	0.008	0.031	0.5	1	1	0.25	2	0.5	>64
**C.**	ATCC 24433	0.031	0.008	0.031	0.5	2	1	0.25	2	0.5	>64
**D.**	ATCC 10231	0.063	0.016	0.063	0.5	2	1	0.25	2	0.5	>64
**E.**	ATCC 90028	0.063	0.016	0.031	1	2	1	0.5	2	0.5	>64
**F.**	DPL1016 (DP-A15-10)	32	4	8	>64	>64	>64	>64	>64	>64	>64
**G.**	T-2068 (DP-C42)	16	4	4	64	64	>64	>64	>64	>64	>64
**H.**	EF1620	0.031	0.008	0.016	0.5	4	2	0.5	4	0.5	>64
**I.**	ATCC 2001	0.063	0.008	0.063	1	2	1	0.5	2	1	>64
**J.**	ATCC 66032	0.063	0.016	0.031	2	4	2	0.5	4	1	>64
**K.**	ATCC 90030	0.125	0.016	0.031	1	4	2	0.5	4	1	>64
**L.**	T-192 (1775)	0.016	0.008	0.016	0.5	2	1	0.25	2	0.5	>64
**M.**	TGL00275	16	4	8	>64	>64	>64	>64	>64	>64	>64
**N.**	TGL00277	>64	>64	>64	>64	>64	>64	>64	>64	>64	>64

[a] (A–G) *C. albicans* strains; (H–N) *C. glabrata* strains; (F, G, M, and N) strains are echinocandin resistant *FKS* mutations.

[b] Non-conjugated Azide-functionalized fluorescent dyes (TMR and NBD) were inactive against the entire panel of *Candida* strains.

2.3. Live cell imaging.

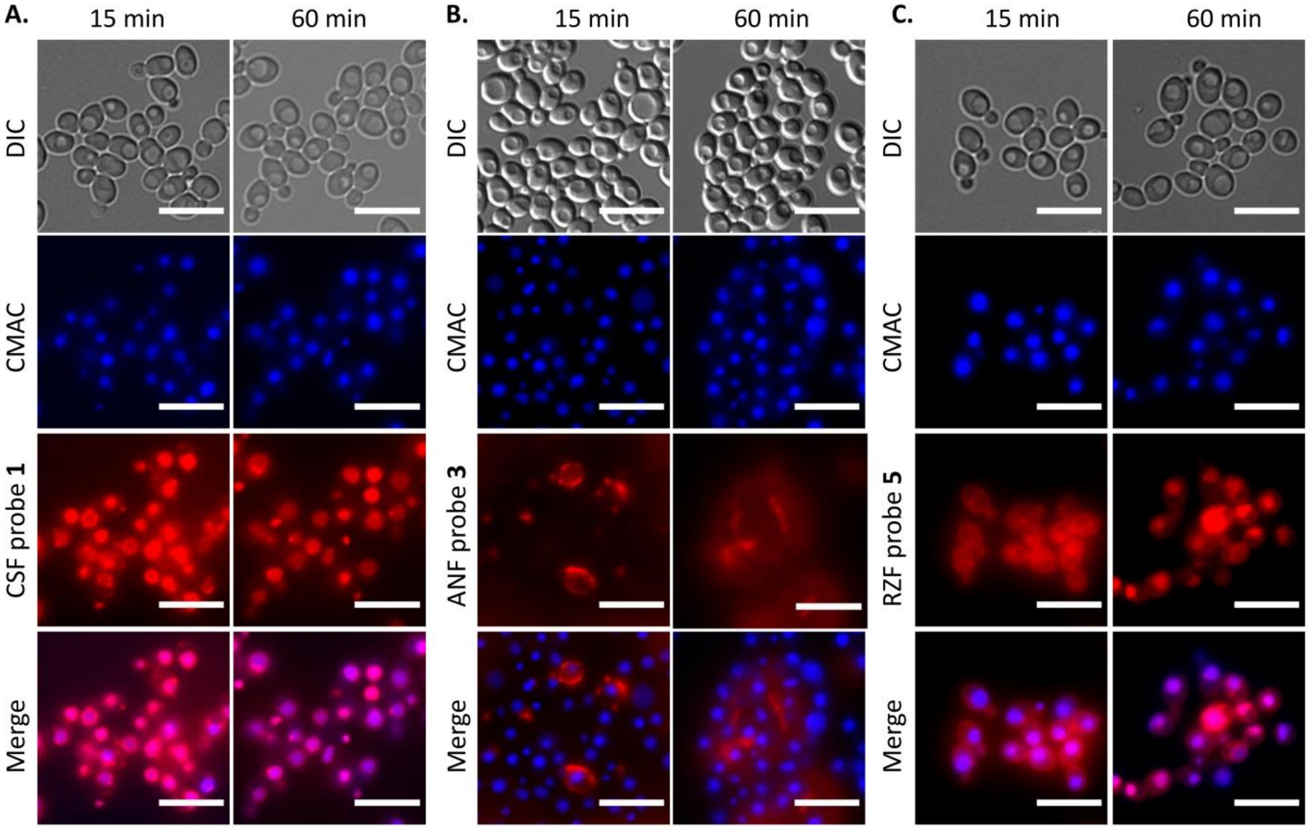

Figure S9. Time-dependent subcellular distribution of TMR-labeled echinocandin probes **1**, **3**, and **5**. Differential interference contrast (DIC) and fluorescent images of *C. glabrata* ATCC 66032 yeast cells incubated for 15 or 60 minutes in PBS with the vacuole-specific fluorescent dye CellTracker Blue CMAC (10 μM, blue) and with: **(A)** CSF probe **1** (1 μM, red); **(B)** ANF probe **3** (1 μM, red); or **(C)** RZF probe **5** (1 μM, red). Scale bars, 10 μm. A bandpass filter with an excitation of 560/20 nm and an emission wavelength of 629.5/37.5 nm was used for TMR. A bandpass filter with an excitation wavelength of 350/25 nm and an emission wavelength of 460/25 nm was used for the vacuole CellTracker Blue CMAC.

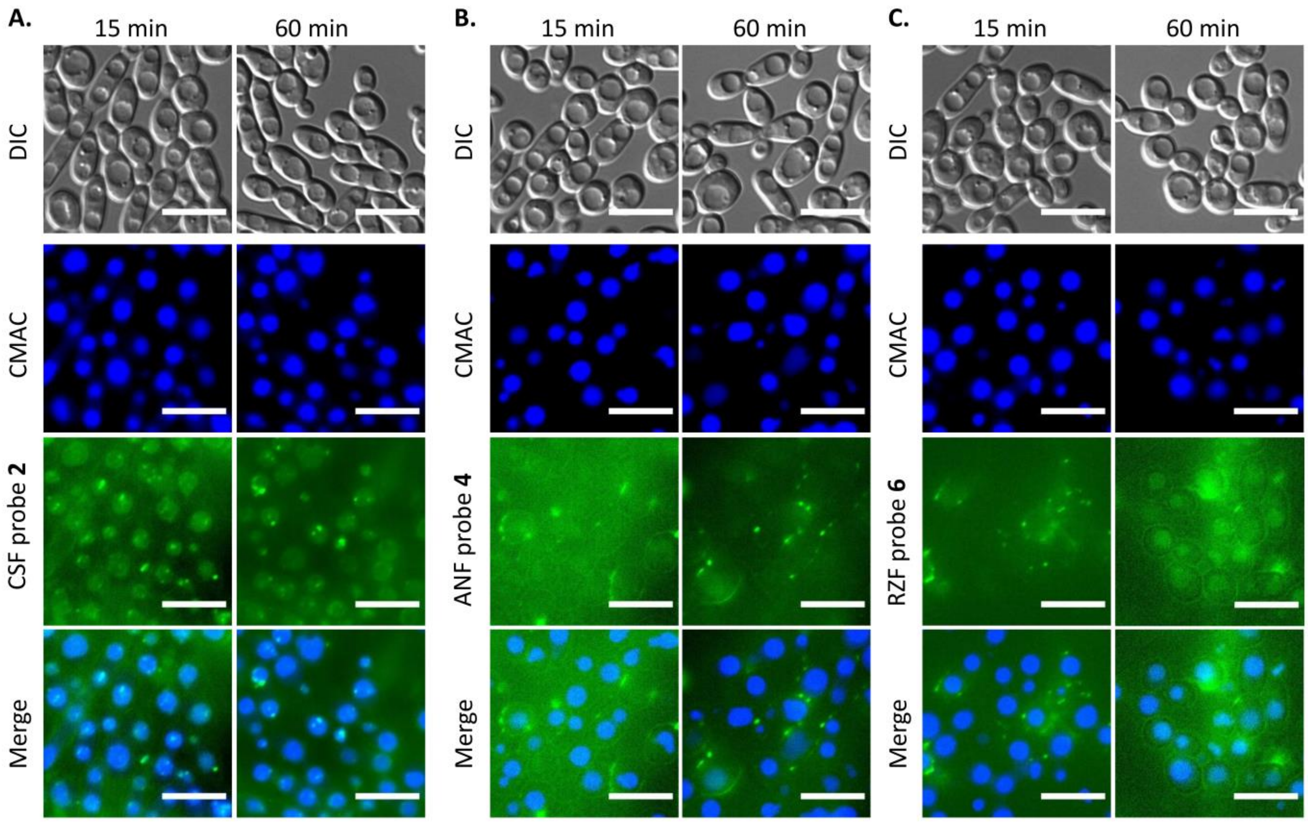

Figure S10. Time-dependent subcellular distribution of NBD-based echinocandin probes **2**, **4**, and **6**. DIC and fluorescent images of *C. albicans* SC5314 yeast cells incubated for 15 and 60 minutes in PBS with the vacuole-specific fluorescent dye CellTracker Blue CMAC (10 μM, blue) and with: **(A)** CSF probe **2** (5 μM, green); **(B)** ANF probe **4** (5 μM, green); or **(C)** RZF probe **6** (5 μM, green). Scale bars, 10 μm. A bandpass filter with an excitation wavelength of 470/20 nm, and an emission wavelength of 525/25 nm was used for NBD. A bandpass filter with an excitation wavelength of 350/25 nm and an emission wavelength of 460/25 nm was used for the vacuole CellTracker Blue CMAC.

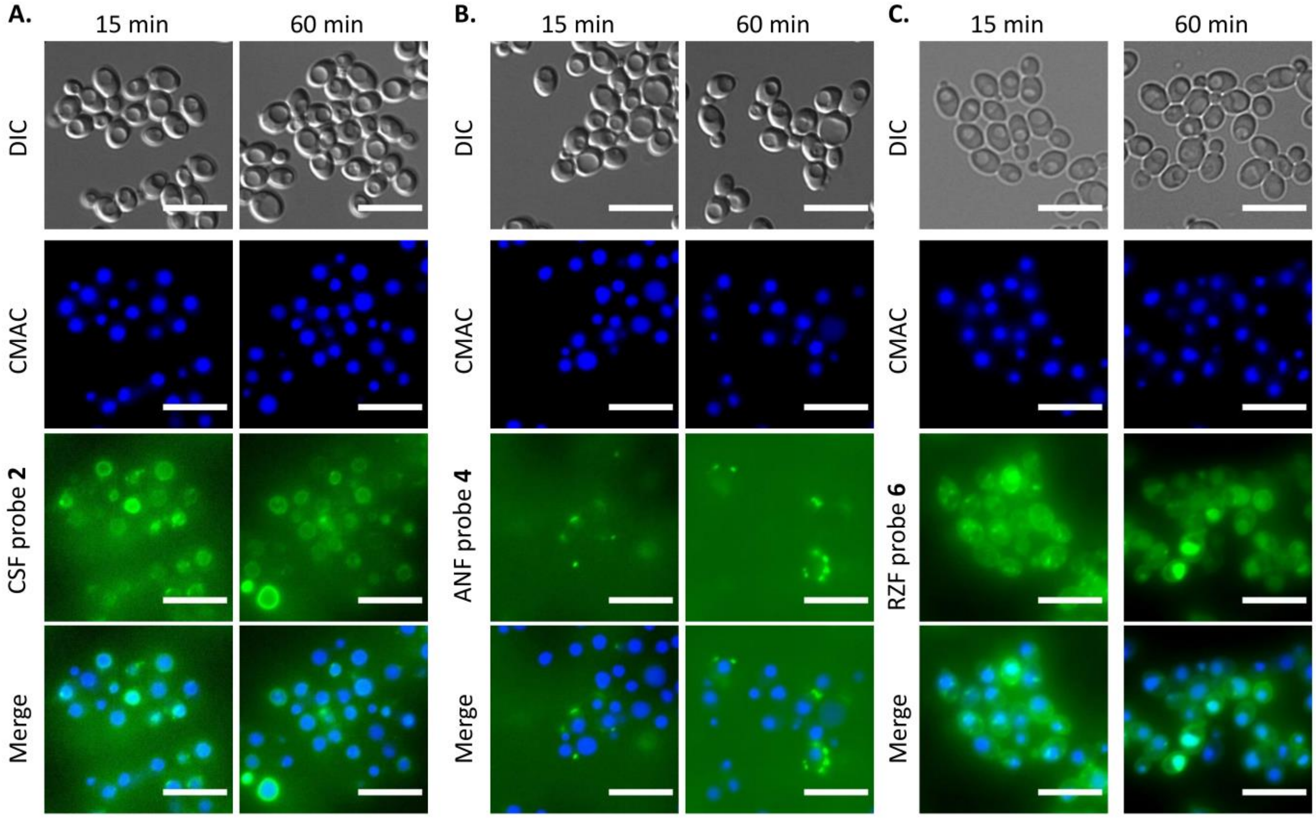

Figure S11. Time-dependent subcellular distribution of NBD-based echinocandin probes **2**, **4**, and **6.** DIC and fluorescent images of *C. glabrata* ATCC 66032 yeast cells incubated for 15 and 60 minutes in PBS with the vacuole-specific fluorescent dye CellTracker Blue CMAC (10 μM, blue) and with: **(A)** CSF probe **2** (5 μM, green); **(B)** ANF probe **4** (5 μM, green); or **(C)** RZF probe **6** (5 μM, green). Scale bars, 10 μm. A bandpass filter with an excitation wavelength of 470/20 nm, and an emission wavelength of 525/25 nm was used for NBD. A bandpass filter with an excitation wavelength of 350/25 nm and an emission wavelength of 460/25 nm was used for the vacuole CellTracker Blue CMAC.

2.4. Measurement of the degradation of echinocandins by *Candida* cells.

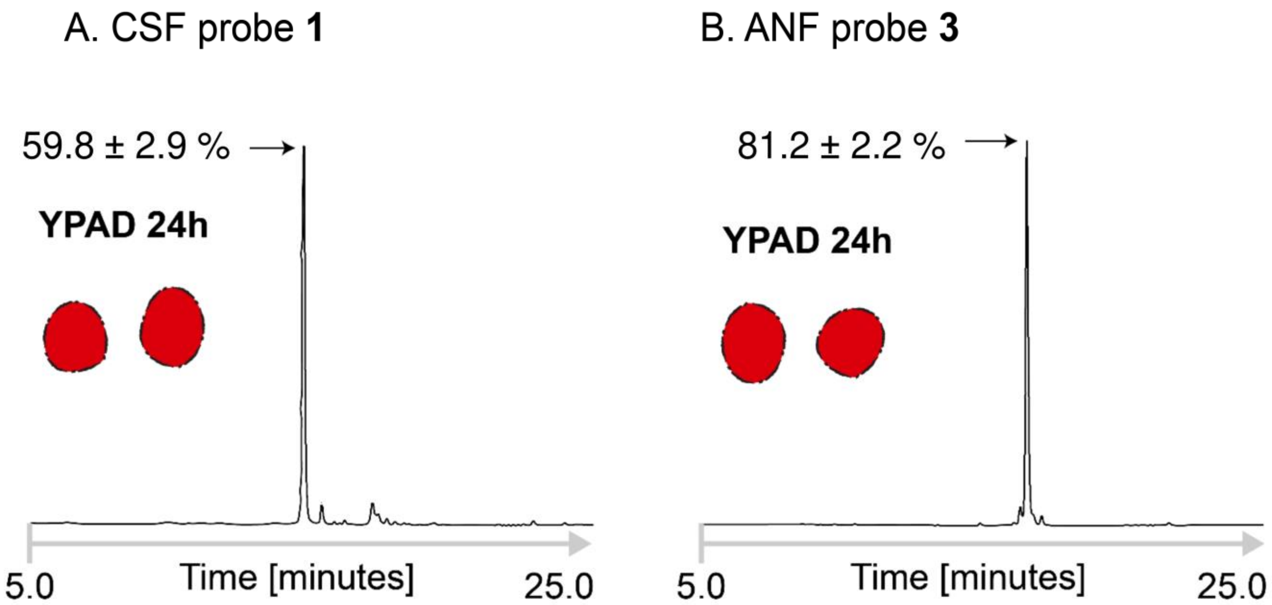

Figure S12. Reverse-phase HPLC analysis of lysates of *C. albicans* SC5314 cells after pre-incubation in YPAD for 24 h with: (A) CSF probe **1** or (B) ANF probe **3**. The illustrated red colored cells indicate that after 2 h of incubation in the nutrient-rich growth media YPAD, most cells were dead and their entire cytoplasm was brightly stained with the TMR-labeled echinocandin probes. Each sample was tested in two independent sets of experiments. The percentages of intact echinocandin are presented as means (two replicates) ± standard deviation.

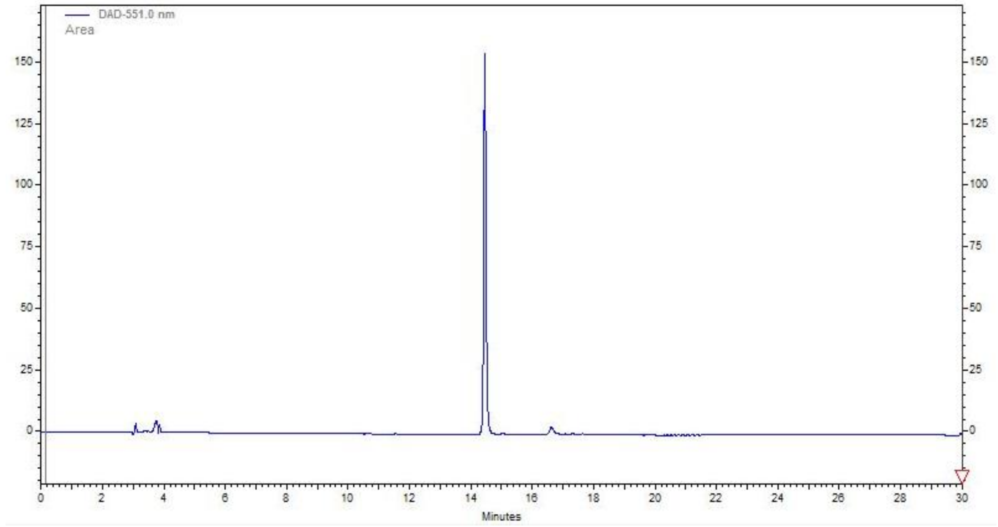

Figure S13. Analytical RP-HPLC analysis of TMR-labeled CSF probe **1** after 24 h incubation in *C. albicans* SC5314 cell lysate. Under these conditions 78% of probe **1** remained intact.

3. NMR spectra

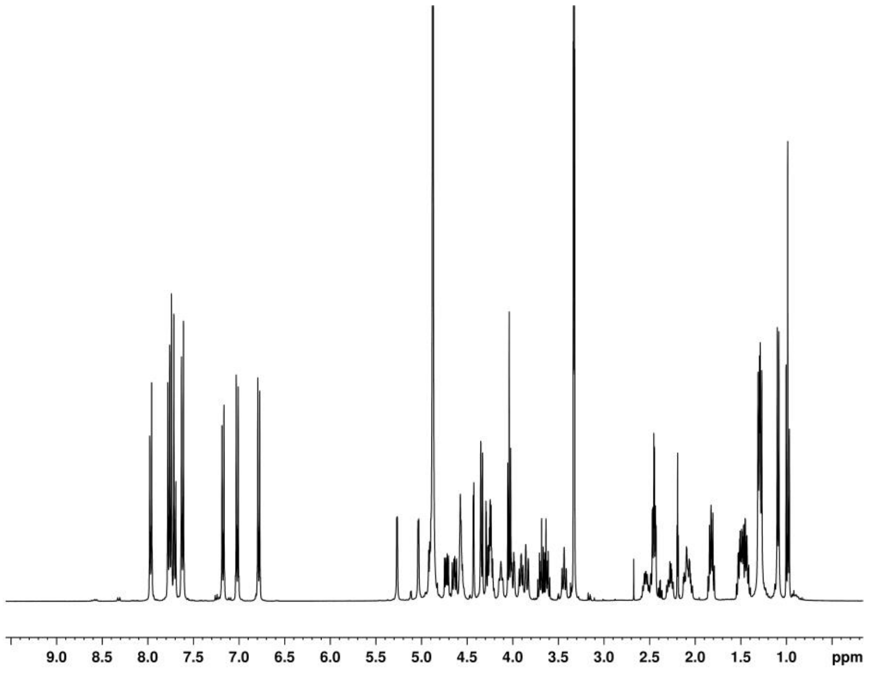

Figure S14. 400 MHz ^{1}H-NMR spectrum of compound **2a** in CD_3OD.

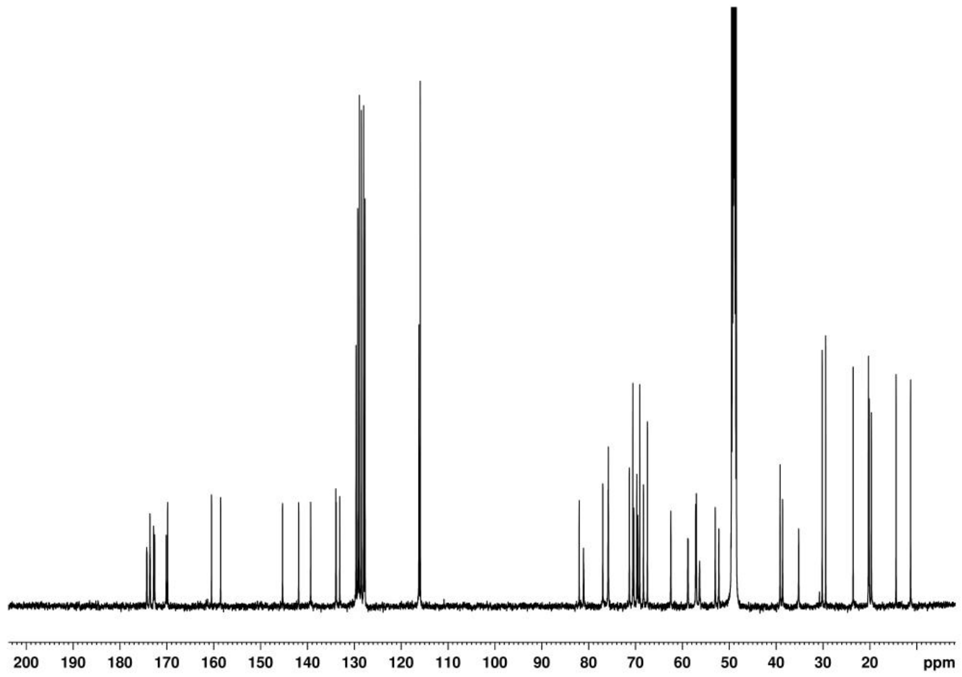

Figure S15. 125 MHz ^{13}C-NMR spectrum of compound **2a** in CD_3OD.

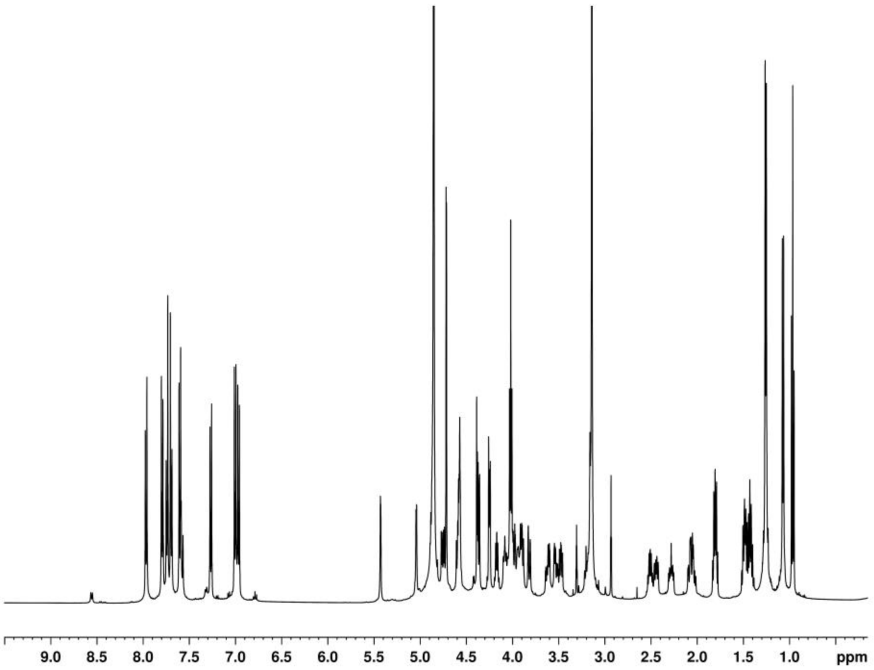

Figure S16. 500 MHz ^{1}H-NMR spectrum of compound **3a** in CD_3OD.

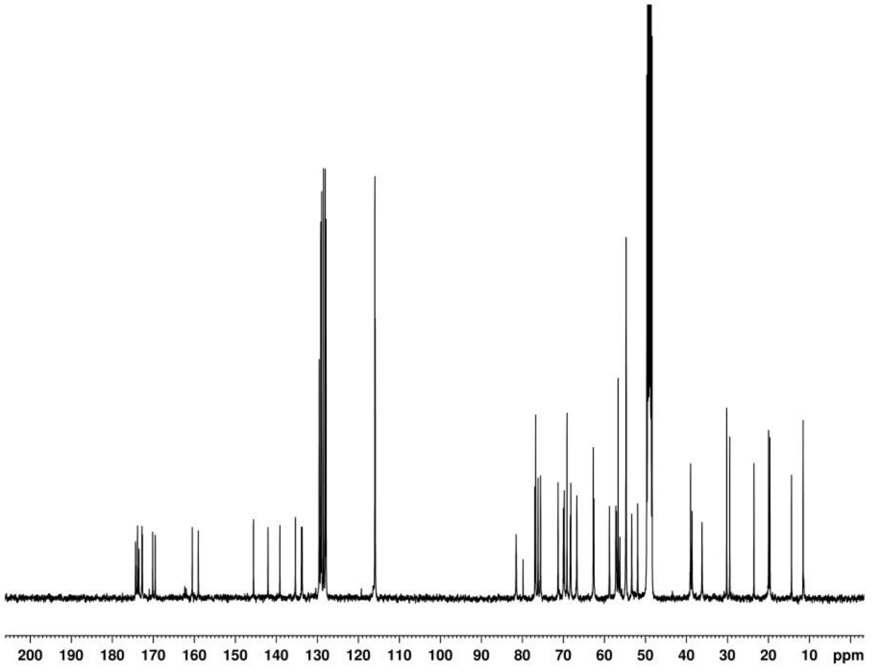

Figure S17. 100 MHz ^{13}C-NMR spectrum of compound **3a** in CD_3OD.

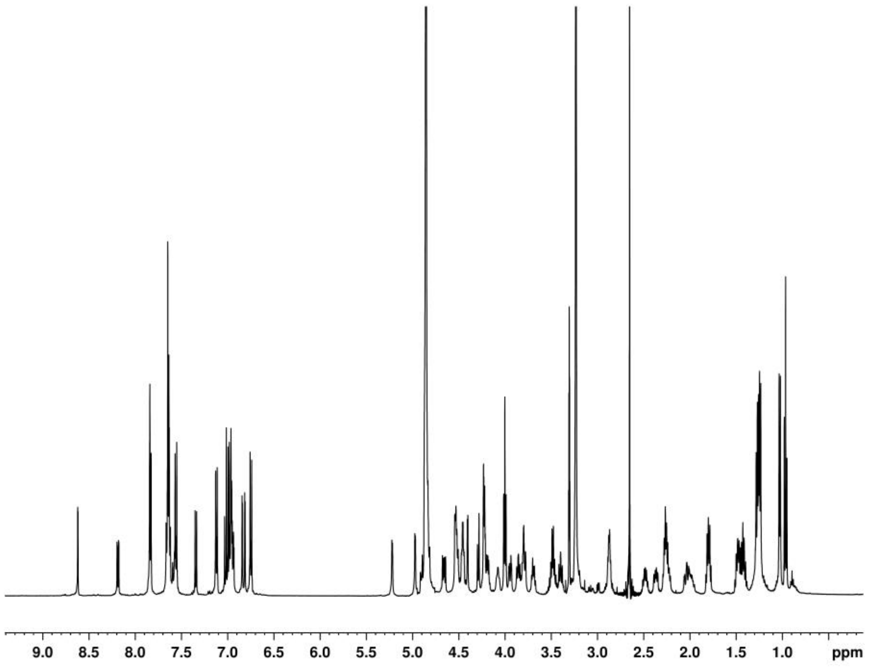

Figure S18. 500 MHz ^{1}H-NMR spectrum of probe **3** in CD_3OD.

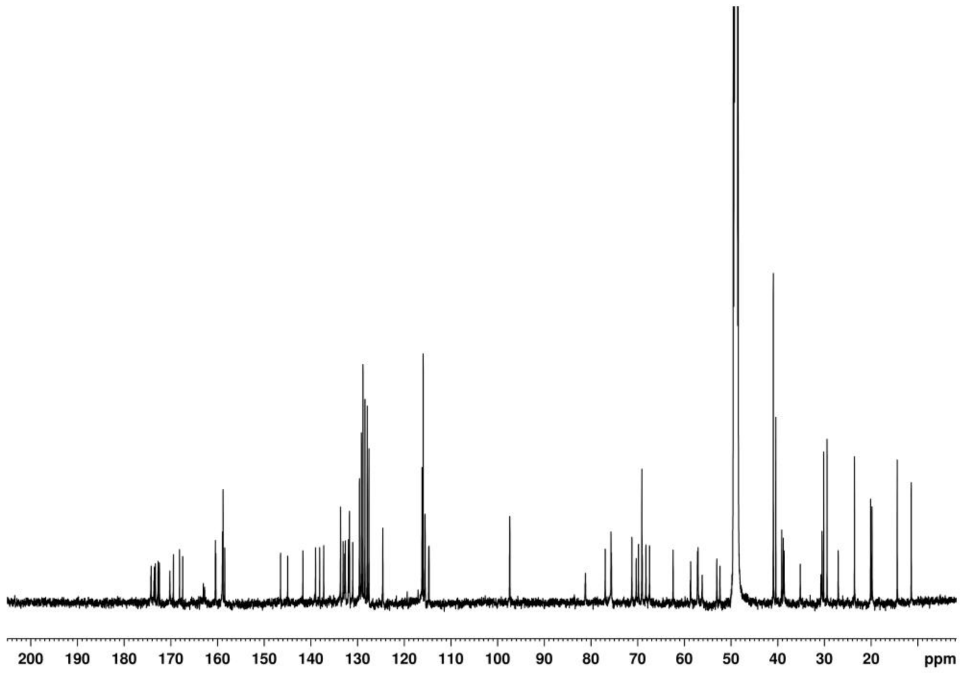

Figure S19. 125 MHz ^{13}C-NMR spectrum of probe **3** in CD_3OD.

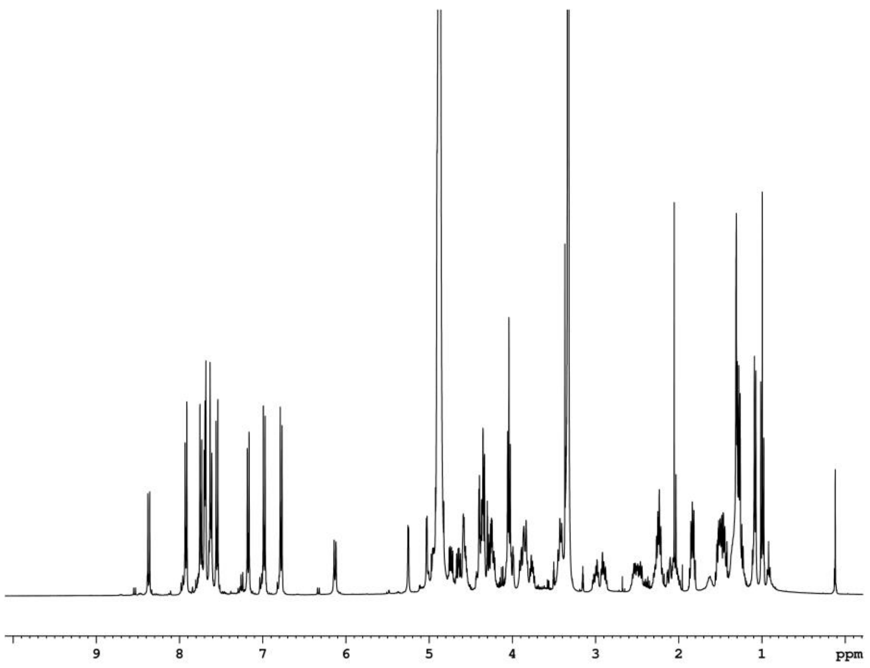

Figure S20. 400 MHz ^{1}H-NMR spectrum of probe **4** in CD_3OD.

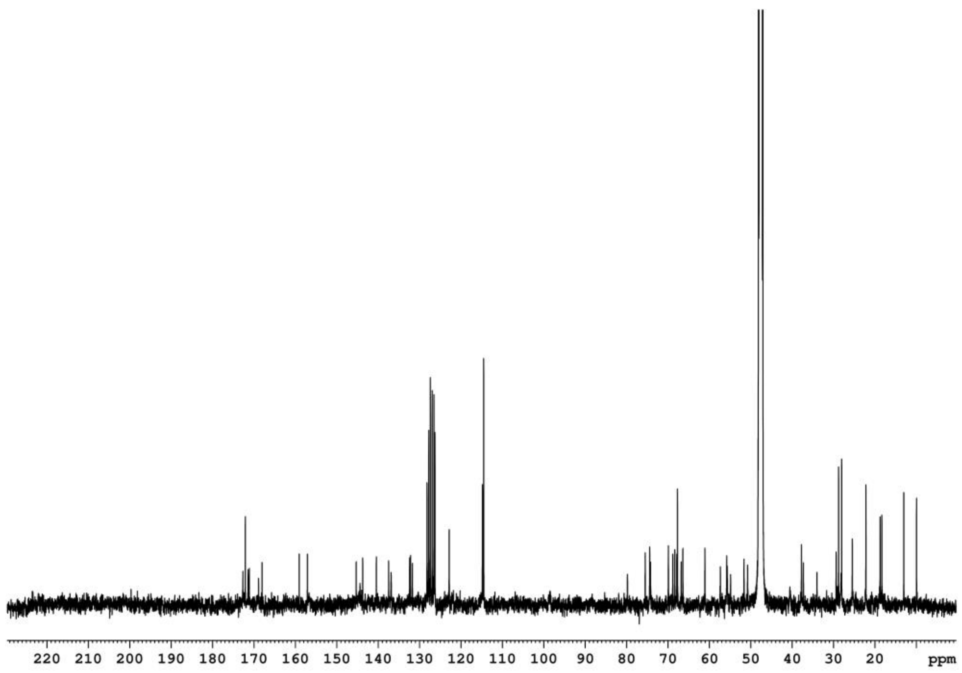

Figure S21. 125 MHz ^{13}C-NMR spectrum of probe **4** in CD_3OD.

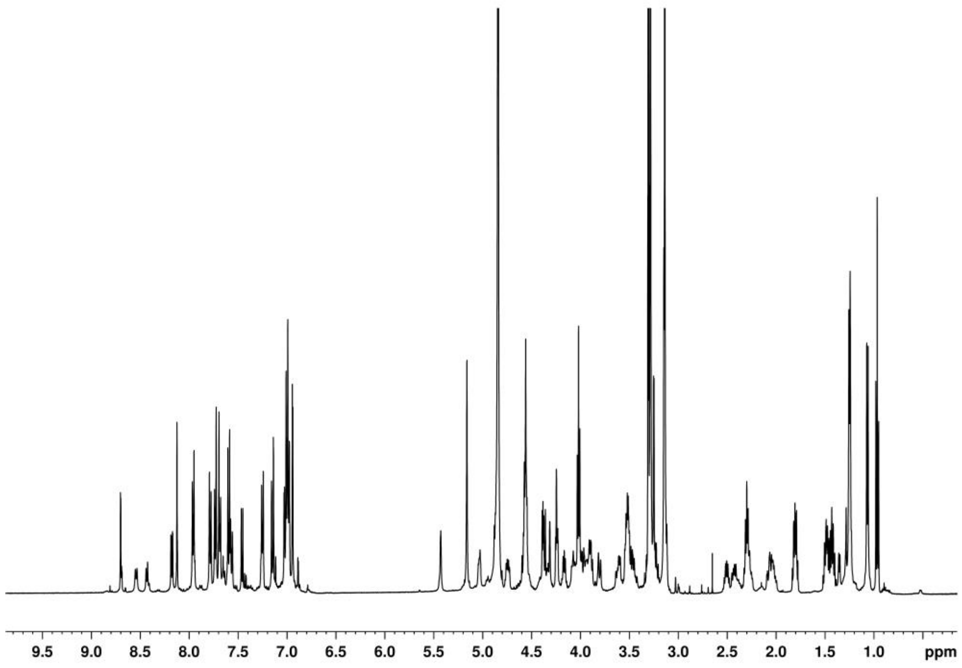

Figure S22. 500 MHz ^{1}H-NMR spectrum of probe **5** in CD_3OD.

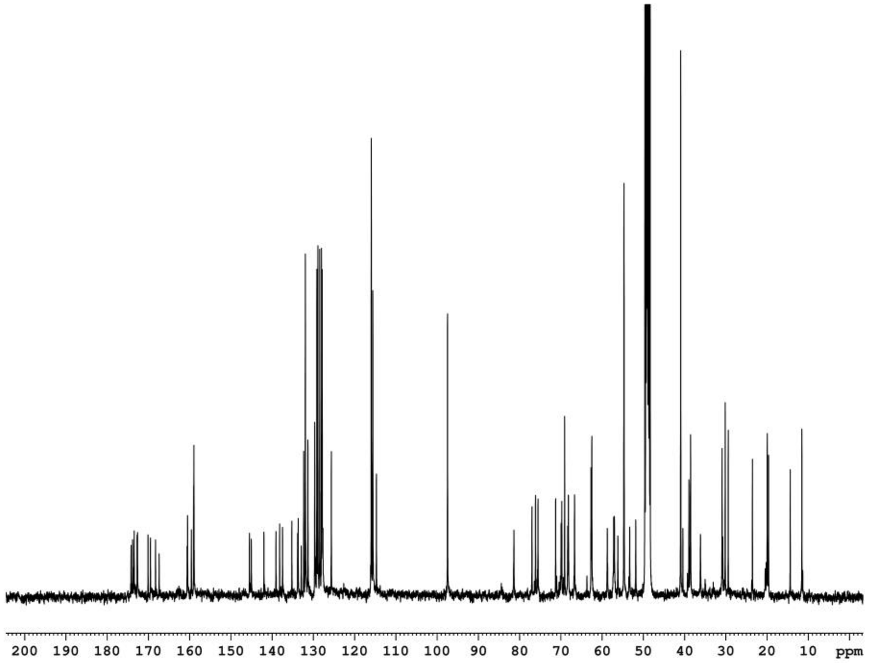

Figure S23. 100 MHz ^{13}C-NMR spectrum of probe **5** in CD_3OD.

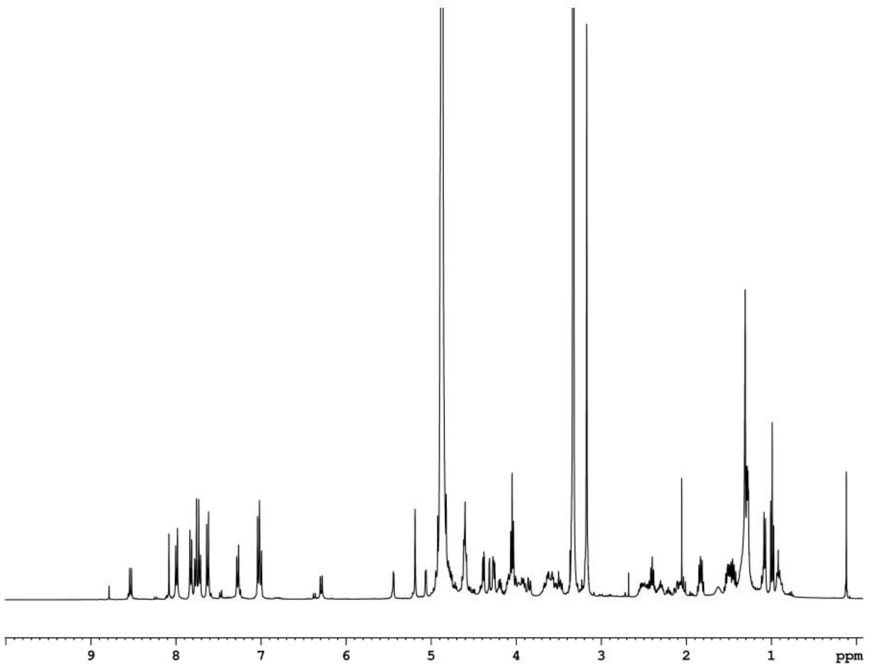

Figure S24. 400 MHz ^{1}H-NMR spectrum of probe **6** in CD_3OD.

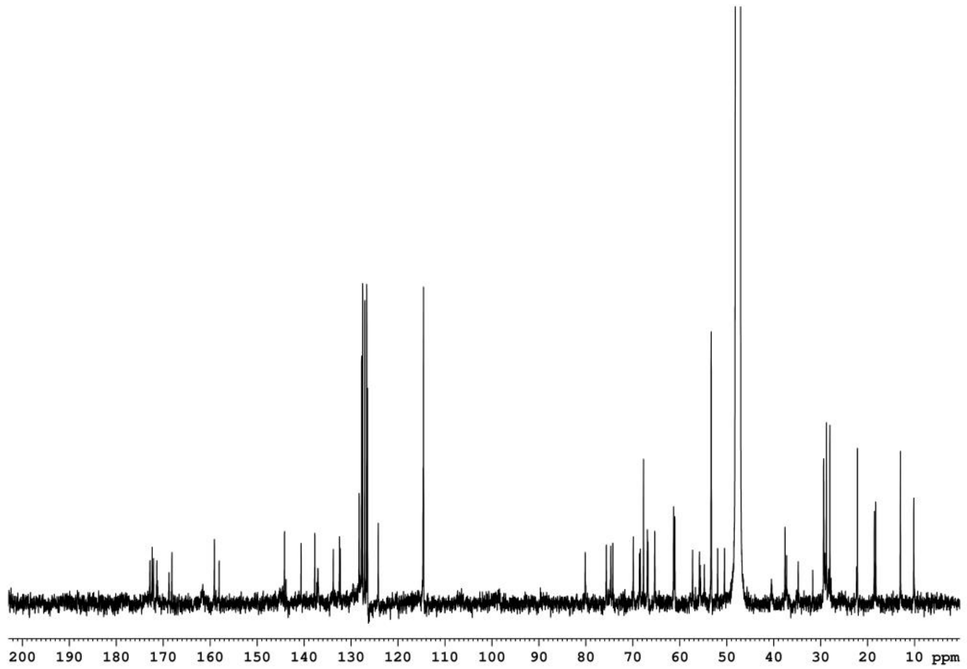

Figure S25. 125 MHz ^{13}C-NMR spectrum of probe **6** in CD_3OD.

4. References

(1) Jaber, Q. Z.; Bibi, M.; Ksiezopolska, E.; Gabaldon, T.; Berman, J.; Fridman, M. Elevated Vacuolar Uptake of Fluorescently Labeled Antifungal Drug Caspofungin Predicts Echinocandin Resistance in Pathogenic Yeast. *ACS Cent. Sci.* **2020**, *6* (10), 1698–1712. https://doi.org/10.1021/acscentsci.0c00813.

(2) J. K. Duke, L. C. Patrick, M. N. Balkrishna, R. Balasingham. Antifungal Agents and Uses Thereof. United States Patent, **2015**, US9217014.

(3) Jones, T.; Federspiel, N. A.; Chibana, H.; Dungan, J.; Kalman, S.; Magee, B. B.; Newport, G.; Thorstenson, Y. R.; Agabian, N.; Magee, P. T.; Davis, R. W.; Scherer, S. The Diploid Genome Sequence of *Candida* Albicans. *Proc. Natl. Acad. Sci. U. S. A.* **2004**, *101* (19), 7329–7334. https://doi.org/10.1073/pnas.0401648101.

(4) Vincent, B. M.; Lancaster, A. K.; Scherz-Shouval, R.; Whitesell, L.; Lindquist, S. Fitness Trade-Offs Restrict the Evolution of Resistance to Amphotericin B. *PLoS Biol.* **2013**, *11* (10), e1001692. https://doi.org/10.1371/journal.pbio.1001692.

(5) Ben-Ami, R.; Garcia-Effron, G.; Lewis, R. E.; Gamarra, S.; Leventakos, K.; Perlin, D. S.; Kontoyiannis, D. P. Fitness and Virulence Costs of *Candida* Albicans FKS1 Hot Spot Mutations Associated with Echinocandin Resistance. *J. Infect. Dis.* **2011**, *204* (4), 626–635. https://doi.org/10.1093/infdis/jir351.

(6) Carreté, L.; Ksiezopolska, E.; Pegueroles, C.; Gómez-Molero, E.; Saus, E.; Iraola-Guzmán, S.; Loska, D.; Bader, O.; Fairhead, C.; Gabaldón, T. Patterns of Genomic Variation in the Opportunistic Pathogen *Candida* Glabrata Suggest the Existence of Mating and a Secondary Association with Humans. *Curr. Biol.* **2018**, *28* (1), 15–27.e7. https://doi.org/10.1016/j.cub.2017.11.027.

(7) Muller, H.; Hennequin, C.; Gallaud, J.; Dujon, B.; Fairhead, C. The Asexual Yeast *Candida* Glabrata Maintains Distinct a and α Haploid Mating Types. *Eukaryot. Cell* **2008**, *7* (5), 848–858. https://doi.org/10.1128/EC.00456–07.

(8) Ben-Ami, R.; Zimmerman, O.; Finn, T.; Amit, S.; Novikov, A.; Wertheimer, N.; Lurie-Weinberger, M.; Berman, J. Heteroresistance to Fluconazole Is a Continuously Distributed Phenotype among *Candida* Glabrata Clinical Strains Associated with in Vivo Persistence. *MBio* **2016**, *7* (4). https://doi.org/10.1128/mBio.00655–16.
